# Transition metal coordination to degradation products in battery electrolytes revealed by NMR and EPR spectroscopy

**DOI:** 10.1039/d5ee01250c

**Published:** 2025-10-29

**Authors:** Jennifer P. Allen, Conrad Szczuka, Erlendur Jónsson, Rüdiger-A. Eichel, Josef Granwehr, Clare P. Grey

**Affiliations:** a Yusuf Hamied Department of Chemistry, University of Cambridge Lensfield Road Cambridge CB2 1EW Cambridge UK cpg27@cam.ac.uk; b The Faraday Institution, Quad One, Harwell Science and Innovation Campus Didcot OX11 0RA UK; c Institute of Energy Technologies (IET-1), Forschungszentrum Jülich GmbH 52425 Jülich Germany; d Institute of Physical Chemistry, RWTH Aachen University 52056 Aachen Germany; e Institute of Technical and Macromolecular Chemistry, RWTH Aachen University 52056 Aachen Germany

## Abstract

The dissolution of transition metals from lithium-ion battery materials contributes to cell failure. Developing a better understanding of transition metal solvation, reactivity, and deposition will help mitigate transition metal dissolution and, thus, facilitate batteries with higher capacity and longer lifetime. In this work, Mn^2+^ and Ni^2+^ coordination in degraded LiPF_6_ carbonate electrolyte solutions is examined *via*^1^H and ^19^F NMR relaxometry at ambient temperatures and pulsed (double resonance HYSCORE and ENDOR) EPR spectroscopy of the frozen solutions. Critically, the solvation spheres of transition metals in heat-degraded electrolytes are shown to differ from those found in pristine electrolytes, with significant coordination to LiPF_6_-derived fluorophosphate degradation species—a factor that will impact the mechanisms and the extent of transition metal dissolution. The Mn^2+^ coordination environment is shown to be affected by adding a variety of species including water, ethylene glycol, and acetylacetonate, increasingly displacing EC and PF_6_^−^ from inner and outer Mn^2+^ coordination shells. EPR and NMR studies of electrolytes containing the battery additive and proposed degradation product LiPO_2_F_2_ explicitly confirm that the transition metals coordinate to PO_2_F_2_^−^ in both the inner and outer sphere. By contrast, in heat-degraded electrolytes, additional protonated (uncharged) fluorophosphate species are also present in the metal solvation shell, as clearly demonstrated *via* large ^1^H and ^31^P hyperfine interactions, seen in the EPR spectra of Mn^2+^ complexes. To probe transition metal deposition, Mn^2+^ and Ni^2+^ were exposed to a wide variety of salts found in the solid electrolyte interphase (SEI), revealing for the first time that the metathesis deposition pathway is viable with many different SEI species.

## Abstract 2

Magnetic resonance studies of battery electrolytes reveal that coordination of metal ions dissolved from cathodes differs between pristine and degraded electrolytes.

## Graphical abstract

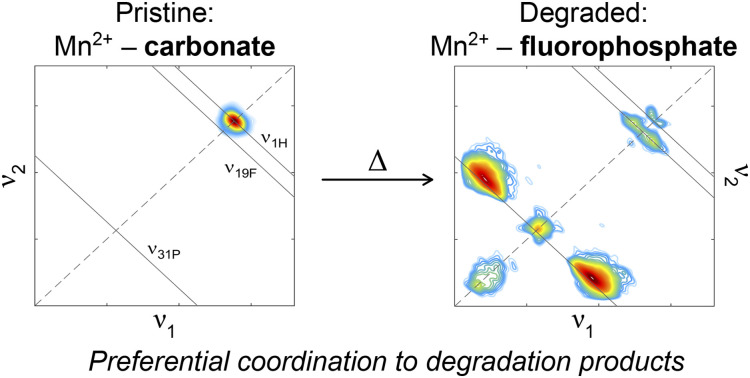

**Broader context**

The dissolution of transition metal ions from essentially all battery cathode active materials is thought to be a significant cause of battery capacity fade. Dissolution can occur as part of cathode degradation processes, and these dissolved transition metals will cross over from cathode to anode, where they may precipitate, potentially contributing to breakdown of the passivating layer that forms on the graphite anode (the solid electrolyte interphase). Surprisingly, little is known about how the transition metals dissolve in battery electrolytes, from the pristine to the more degraded solutions that are representative of practical systems. In order to develop strategies to mitigate dissolution, including the design of new additives, coatings, and electrolytes, a fundamental understanding of how the relevant transition metals are dissolved in both pristine and degraded electrolytes is required. This paper attacks the issue directly by developing new magnetic resonance approaches to study these electrolytes and shows clearly that the coordination of these ions in degraded electrolytes is very different from coordination in the pristine electrolytes, and that these ions are strongly coordinated to a series of anions often found in degraded electrolytes and used as additives.

## Introduction

While the dissolution of transition metals from battery cathode active materials is thought to cause significant battery capacity fade *via* a myriad of different mechanisms,^1–6^ comparatively little work has been performed to study the solvation of the transition metals once they are dissolved in the battery electrolyte solution.^7–11^ Even less attention has been paid to how the solvation may differ between ideal pristine electrolyte solutions and (partly) degraded electrolyte solutions that more accurately represent the use case, particularly in regimes where the rate of transition metal dissolution is enhanced. Although an understanding of the transition metal solvation shell is not generally directly relevant to the bulk properties of the electrolyte solution, transition metal solvation and desolvation are important for transition metal dissolution and deposition behaviour, respectively. For example, a study of metal dissolution in LiNi_0.5_Mn_1.5_O_4_/graphite cells proposed that solvent oxidation at the cathode surface generates β-diketonate species that are adsorbed on the surface, chelating the transition metals and facilitating metal dissolution.^12^ Another study proposed that the chelation of dissolved Mn^2+^ by unspecified electrolyte degradation products may create neutral Mn complexes that can be transported through the outer, organic layer of the solid electrolyte interphase (SEI) and deposited at the surface of the inner, inorganic SEI.^13^ One density functional theory (DFT) study showed that P–F bonds in PF_6_^−^ were longer in the presence of Mn^2+^, facilitating the formation of MnF^+^ and the reactive Lewis acid, PF_5_.^8^ In combination with experiments showing severe discolouration of electrolyte solutions stored with Mn^2+^,^8,14^ this suggests that dissolved transition metals may actually contribute to bulk electrolyte decomposition reactions even before their deposition at the anode occurs.

The process of transition metal dissolution may be driven by or coupled with electrolyte decomposition reactions occurring at the cathode; for instance, acidic oxidation or hydrolysis products may leach transition metals from the cathode.^12,15–20^ Thus, when transition metal dissolution occurs, it is likely that the electrolyte solution is already degraded to some degree, and it is therefore vital to consider how transition metals are then solvated in the degraded electrolyte solution. However, computational studies of transition metal coordination in battery electrolyte solutions have largely focused on the components of the pristine electrolyte.^7,8^ One simulation study of Mn^2+^ dissolution processes incorporated ethylene carbonate (EC) decomposition products and F^−^ on the modelled LiMn_2_O_4_ (LMO) surface, but simulated the electrolyte solution as 32 EC molecules.^21^ Notably, modelling studies that have incorporated coordinating degradation species have shown that they do have an effect: one study incorporated F^−^ into a diethyl carbonate solution and found that F^−^ may facilitate the dissolution of Ni and Mn from LiNi_0.5_Mn_1.5_O_4_ (LNMO);^22^ another simulation study found that biacetyl complexation at the surface of LiNi_*x*_Mn_*y*_Co_1−*x*−*y*_O_2_ (NMC) increased Ni dissolution, which was then confirmed experimentally.^23^ This is further supported by an experimental study showing enhanced dissolution from charged LiNi_0.5_Mn_0.3_Co_0.2_O_2_ (NMC532) cathodes stored with a solution containing the salt lithium acetylacetonate (Li(acac)), as compared to the control electrolyte containing LiPF_6_.^24^ Another study found that transition metals dissolved from delithiated cathode materials in the presence of carbonate solvents tended to coordinate to anionic species.^10^*Operando* studies have shown that exposure of cathode materials to water-contaminated electrolyte solutions enhanced transition metal dissolution,^25^ while exposure to thermally degraded electrolyte solutions did not appear to enhance transition metal dissolution, complicating the picture.^26^ If transition metals are indeed coordinated to degradation products in solution, whether thermally, chemically, or electrochemically generated, then models of how dissolution (solvation) and deposition (desolvation) occur may benefit from the incorporation of soluble electrolyte decomposition species in the simulated electrolyte solution. Although simulation of additional components is increasingly computationally expensive, targeted experiments may serve as a basis for computational work by identifying the solution species that are most likely to affect transition metal dissolution.

The role of PF_6_^−^ degradation products in battery chemistry is complex. While alkylfluorophosphates have been proposed to trigger autocatalytic decomposition of the electrolyte solution,^27^ and while difluorophosphate generated *in situ* may serve as an indicator of PF_6_^−^ hydrolysis,^28–31^ difluorophosphate itself does not appear to be harmful in cells: studies using a small percentage of added LiPO_2_F_2_ in the electrolyte solution have shown that it enhances cell performance^32–38^ by modifying the SEI at the cathode^33,34,37,38^ and anode^34,36,37^ surfaces, offering protective benefits, such as minimising impedance growth. With respect to transition metal dissolution, previous work has shown that added LiPO_2_F_2_ results in lower levels of transition metals deposited at the negative electrode.^32–35^ It has been proposed that the PO_2_F_2_^−^ additive may decompose into PO_4_^3−^ and PO_3_F^2−^, which may induce precipitation of dissolved metals before they migrate to the anode (presumably at the cathode or in the separator).^32,35,39–41^

Another pathway to prevent transition metal deposition, rather than inducing precipitation of transition metals before they reach the anode, involves sequestering them in solution. Chelating agents have been added to cells to prevent the deposition of dissolved metals by modifying the electrolyte solution, separator, or anode. In the electrolyte solution, transition metals have been sequestered by using crown ether,^14^ aza-crown ether,^42^ or 1,2-bis(diphenylphosphino)ethane^43^ additives, or a specialised chelating polymer gel electrolyte.^44^ Highly concentrated electrolyte solutions have been suggested to prevent the deposition of dissolved Mn^2+^ by increasing the formation of contact ion pairs and aggregates, in turn decreasing the favourability of incorporation in the SEI.^9^ Numerous functionalised separators have been employed to trap dissolved transition metals in cells, with varying degrees of selectivity and efficiency,^45,46^ and at the negative electrode, an aza-crown ether functionalised binder has been proposed.^42^ Chelating agents have also been used in the cathode to coordinate the metals and prevent dissolution into the electrolyte solution, for example, by functionalising the cathode binder^47–50^ or conductive carbon.^51^ The effects of transition metal chelation in lithium-ion cells are complex, and it may be beneficial or harmful, depending on the context. Storage with 3 : 7 ethylene carbonate : ethyl methyl carbonate (EC : EMC, w/w) solution containing lithium acetylacetonate, Li(acac), has been shown to enhance Mn and Co dissolution (not Ni dissolution) from charged NMC532, although dissolution from pristine cathodes was unaffected.^24^ However, acetylacetone has also been used to chelate Mn and prevent its dissolution from LMO spinel: rinsing electrode materials with an acac solution and then sintering the materials was found to reduce Mn dissolution.^52,53^ This strategy succeeded because the acac solution leached Mn from the LMO surface, creating a Mn-deficient surface layer that was less prone to dissolution.^52,53^ These results suggest that an understanding of chelation is important and can directly be used to prevent cell degradation.

Generally, it is not entirely clear what fraction of transition metals remains in the electrolyte as soluble complexes and what fraction precipitates at the cathode or deposits at the anode, and how this is affected by the species present in solution, motivating further studies. In a cell, where many species are present simultaneously, it is difficult to predict how the formation of soluble chelate complexes may impact metal deposition. Both strategies outlined above, of inducing transition metal precipitation at the cathode and of sequestering metal ions in solution, target the prevention of metal deposition at the anode, and both strategies require an understanding of transition metal solvation, complexation, and desolvation behaviours.

In this work, we study the coordination of Mn^2+^ and Ni^2+^ in a degraded carbonate-based electrolyte. These species are of interest because Mn^2+^ is thought to be more harmful to cells than other dissolved metals,^3,5,54–56^ and Ni^2+^ is the most abundant metal in nickel-rich lithium-ion battery cathode materials; both ions represent typical divalent^5,20,57–60^ paramagnetic dissolved transition metal ions. Mn^2+^ and Ni^2+^ species are studied separately in this work, such that results may be applicable to any chemistry containing either ion, including NMC, LMO, LNMO, LiNi_*x*_Co_*y*_Al_1−*x*−*y*_O_2_ (NCA), LiNiO_2_ (LNO), LiMn_*x*_Fe_1−*x*_PO_4_ (LMFP), *etc.* The paramagnetism of these ions can be exploited to examine interactions between electron and nuclear spins, *i.e.*, hyperfine interactions, using the complementary techniques of electron paramagnetic resonance (EPR) and nuclear magnetic resonance (NMR) spectroscopy, as illustrated in our previous publication on pristine electrolytes.^11^ Pulsed EPR identifies nearby magnetically-active nuclei through their effect on the paramagnetic centre's electronic spin states, whereas paramagnetic NMR probes the nuclear spins’ relaxation driven by nearby paramagnetic centres. Since the nuclear spins relax on a slower timescale than electron spins, the electron–nuclear (hyperfine) interactions can be studied *via* pulsed EPR experiments. These hyperfine interactions are described by interaction tensors represented through the coupling constants *A*_*x*_, *A*_*y*_, and *A*_*z*_. A non-zero isotropic component (*A*_*x*_ + *A*_*y*_ + *A*_*z*_)/3 indicates electron spin density at the position of the nucleus, generally implying that an inner sphere (bonded) complex is formed, and an anisotropic part describes all through-space dipolar contributions from both inner and outer sphere complexes. Cryogenic temperatures are typically required for pulsed EPR experiments, resulting in a loss of dynamic information, but instead allowing the static compositions and conformations of the transition metal complexes to be determined. EPR studies of the Ni^2+^ ion with its *S* = 1 electron spin are, however, difficult due to its rapid electronic relaxation.^61^ Paramagnetic NMR spectroscopy complements the EPR method because it can be carried out at ambient temperatures, encoding information on dynamics, and may be used to indirectly characterise the solvation shell of most paramagnetic transition metals, with direct relevance here for Ni^2+^.^62^

The coordination sphere of Mn^2+^ and Ni^2+^ in degraded carbonate-based electrolytes is approached in two ways in this work. First, transition metal-containing electrolytes are heated and subsequently examined by pulsed EPR and NMR spectroscopy. Second, transition metal coordination to externally added species is investigated, including difluorophosphate,^28–32^ water,^10,63–67^ acac^−^,^12^ and ethylene glycol (EG),^68–70^ because these and related species have been proposed to form, and/or coordinate to transition metals, in degraded battery electrolytes. EG is a hydrolysis product of EC,^68,69^ and some of the authors have previously proposed the dianion's role in vanadyl coordination in V^4+^-containing solutions.^70^ Lithium difluorophosphate is also commonly used as an electrolyte additive or co-salt.^37,71^ In pristine electrolytes, the transition metal solvation shell is largely comprised of EC;^7,8,11^ however, we show herein by using a combination of EPR and NMR spectroscopies that the solvation shell differs noticeably in degraded electrolytes. Neutral degradation species such as water and ethylene glycol appear to displace ethylene carbonate, while some PF_6_^−^ remains, largely in the outer solvation shell, at ambient temperature for charge balance. Anionic species such as PO_2_F_2_^−^, which was observed to form upon heating of electrolyte solutions, and acac^−^ appear to have a strong binding affinity and displace both EC and PF_6_^−^ from the Mn^2+^ coordination shell. The effect of dissolved Mn^2+^ toward enhancing electrolyte decomposition, which has been previously proposed,^8,14^ is then studied with NMR spectroscopy.

We use heat-treated electrolyte solutions to simulate electrolyte degradation, rather than sourcing cycled electrolytes from lithium-ion cells for the following key reasons. Firstly, using electrolytes from lithium-ion cells would cause various changes to the electrolyte composition that could have significant effects on the measured paramagnetic relaxation enhancement. The most notable of these is, of course, transition metal dissolution, both from the cathode in the lithium-ion cell and the cell casing.^72^ It would not be possible in this way to obtain an electrolyte that contained only Mn^2+^ or only Ni^2+^. Furthermore, electrolyte degradation reactions in the cell would result in changes to the salt concentration, solvent species, and viscosity that would in turn skew the baseline relaxation. The final reason is a practical limitation of the electrolyte volume: NMR relaxometry experiments used 500 μL of undiluted electrolyte (rather than a small volume of electrolyte in a large volume of deuterated solvent), preventing the use of electrolytes extracted from small-scale cells. Had many cells been used to gather a sufficient quantity of degraded electrolyte for all NMR experiments, there would be differences in the electrolyte extracted from each cell, leading to inconsistency between samples. In this case, it was our judgement that adding pristine electrolyte to a sealed container (a closed system) and heating it shows that it is specifically and necessarily the generation of degradation products that is affecting the transition metal solvation, which is later explored through the addition of LiPO_2_F_2_. Nevertheless, building on the insights obtained from the results described below, future studies will focus on incorporating electrochemically-degraded electrolyte solutions sourced from full cells.

Lastly, we explore the deposition of transition metals in the SEI by metathesis, a mechanism that has been suggested for dissolved Mn^2+^,^73–76^ Ni^2+^,^77,78^ and Co^2+^;^79^ even Li^+^ is known to exchange with other Li^+^ in the SEI.^80^*Ex situ* ion exchange experiments have shown by ion chromatography that Li_3_PO_4_ addition^39^ and Na_2_PO_3_F addition^40^ cause precipitation of Mn^2+^, Ni^2+^, and Co^2+^ from electrolyte solutions. Inductively coupled plasma mass spectrometry (ICP-MS) exchange experiments have shown Mn^2+^ precipitation from electrolyte solutions upon addition of Li_2_O, LiF, and Li_2_CO_3_;^75^ Mn^2+^ exchange with the latter two salts is further supported by XPS analysis.^74^ Thus, the effect of a large series of lithium salts—some of which have been shown to be present in the SEI—on transition metal solubility was investigated in this work. Specifically, we examine the precipitation rates of Mn^2+^ and Ni^2+^ using inductively coupled plasma optical emission spectroscopy (ICP-OES) following exposure to 12 different organic and inorganic lithium salts.

## Materials and methods

### Preparation of electrolyte solutions

The bis(trifluoromethane)sulfonimide (TFSI) salts Mn(TFSI)_2_ (Solvionic, 99.5%) and Ni(TFSI)_2_ (Alfa Aesar, ≥97%) were used to model transition metals dissolved from battery cathodes. EPR samples used concentrations of 8 mM Mn(TFSI)_2_ and NMR samples used concentrations of 1 mM Mn(TFSI)_2_ and 1 mM Ni(TFSI)_2_, except where specified otherwise. The main electrolyte solution used in this work comprised 1 M LiPF_6_ in 3 : 7 ethylene carbonate : ethyl methyl carbonate (EC : EMC, v/v). For NMR experiments, this was sourced premixed (soulbrain MI PuriEL R&D 280); for EPR experiments, all solutions were mixed in-house using LiPF_6_ (Sigma Aldrich, ≥99.99% trace metals basis), EC (Sigma Aldrich, 99%, anhydrous), and EMC (Sigma Aldrich, 99%). Owing to the different electrolyte sources, it is likely that EPR samples contained more water impurities than the NMR samples. EPR experiments also used 1 M LiTFSI (Alfa Aesar, 98+%) in 3 : 7 EC : EMC, mixed in-house. To mimic electrolyte degradation, samples were heat-treated (at either 35 °C or 45 °C for EPR samples and 60 °C for NMR samples) or underwent addition of LiPO_2_F_2_ (American Elements, 99%, recrystallised in-house), lithium acetylacetonate (Li(acac), Sigma Aldrich, 97%), distilled H_2_O/D_2_O (Sigma Aldrich, 99.9 atom%), and ethylene glycol (Sigma Aldrich, 99.8%, anhydrous). Additionally, deuterated dimethyl sulfoxide (d_6_-DMSO, Sigma Aldrich, NMR-grade), methanol (MeOD, Sigma Aldrich, NMR-grade), and acetonitrile (CD_3_CN, Fluorochem and Sigma Aldrich, NMR-grade), were added to some EPR and NMR samples to probe metal coordination to these species. All solutions were prepared under argon and all salts were dried under dynamic vacuum at 100 or 110 °C.

### EPR spectroscopy

Mixing, storage, and heat-treatment of electrolyte solutions was performed in Eppendorf tubes made from polypropylene, which is HF-resistant. Solutions were transferred into 2 mm outer diameter (O.D.) quartz EPR tubes (Wilmad, CFQ) for X-band, and 0.9 mm O.D. Suprasil tubes (Wilmad) for Q-band experiments. Tubes were sealed and transferred from the glovebox into the pre-cooled EPR resonator within 15 min after preparation. A Bruker ElexSys E580 spectrometer equipped with an EN4118X-MD4 resonator for X-band and an EN5107-D2 resonator for Q-band frequencies was used for experiments at a temperature of 20 K. Microwave amplification was performed with a 1 kW travelling-wave tube (TWT) pulse amplifier at X-band and with a 150 W TWT at Q-band; radiofrequency amplification was done with a 150 W amplifier.

Field-swept pulsed EPR spectra were obtained using the Hahn echo pulse sequence π/2-*τ*-π-*τ*-echo and a two-step phase cycle. For X-band, *τ* = 200 ns and pulse durations *τ*_π/2_ = 12 ns and *τ*_π_ = 24 ns; for Q-band, *τ* = 160 ns and pulse durations *τ*_π/2_ = 20 ns and *τ*_π_ = 40 ns.

Hyperfine sublevel correlation^81^ (HYSCORE) spectra were recorded using the sequence π/2-*τ*-π/2-*t*_1_-π-*t*_2_-π/2-*τ*-echo with *τ* = 80, 120 ns, *τ*_π/2_ = 12 ns, and *τ*_π_ = 24 ns. Multiple *τ*-values were used to remove blind spot artifacts and *t*_1_, *t*_2_ were incremented by 20 ns starting from 80 ns. A four-step phase cycle was applied. Two-dimensional time traces were baseline-corrected with a third-order polynomial, apodised with a Hamming window function, and zero filled to 1024 points in each dimension, followed by 2D Fourier transformation and calculation of the absolute value. Davies-type electron nuclear double resonance (ENDOR) experiments were conducted at Q-band using the pulse sequence π–D–π/2-*τ*-π-*τ*-echo with *t*_π/2_ = 100 ns, *t*_π_ = 200 ns, and *τ* = 450 ns. During the delay *D*, an rf π-pulse lasting 10 μs was applied.

### DFT calculations and EPR spectra simulation

Density functional calculations were conducted using ORCA 5.0.1.^82^ Structures were geometry optimised with the hybrid functional TPSSh,^83,84^ the zeroth-order regular approximation (ZORA),^85,86^ and def2-TZVP(-f) basis sets^87^ in their ZORA-recontracted version.^88^ Decontracted def2/J auxiliary basis sets were employed for the resolution-of-identity and chain-of-spheres approximation. The convergence criterion was set to tight self-consistent-field. Zero-field splitting and hyperfine coupling tensors were calculated using the hybrid functional PBE0^89^ and ZORA. ZORA-def2-TZVP(-f) basis sets were modified through full s shell decontraction and addition of three steep Gaussians, as explained elsewhere.^90,91^ Spin–orbit coupling was calculated using the spin–orbit mean-field approximation (SOMF(1X)).^92^ Integration accuracy was set to defgrid3 and radial accuracy IntAcc to 11 for manganese and 9 for all other atoms.

EPR spectra were simulated using EasySpin v6.0.0-dev.49^93,94^ running in Matlab v2021b (MathWorks). HYSCORE spectra were simulated considering all magnetic nuclei with hyperfine tensors as obtained from DFT calculations using the simulation module saffron. Second-order perturbation theory and the product rule were used. Input parameters were set as used experimentally and electronic relaxation times as *T*_1e_ = 80 μs and *T*_2e_ = 0.4 μs, which are in the range of those experimentally determined. Post-processing was equivalent as for experiments. ^1^H powder Davies ENDOR spectra were simulated at Q-band frequencies assuming one coupled ^1^H nucleus using the simulation module salt. The obtained spectra were multiplied with the Davies ENDOR detection function.^95,96^

### NMR spectroscopy

^1^H and ^19^F NMR relaxation measurements of electrolyte solutions were performed at ∼25 °C on a Bruker Avance III HD 300 MHz spectrometer using a Bruker double-channel MicWB40 probe. NMR spectra were collected on a Bruker Avance III HD 500 MHz spectrometer using a BBO probe, with a sealed capillary of C_6_D_6_ added to the NMR tube for field locking. No deuterated solvents were used for measurements at 300 MHz. Spin–lattice *T*_1_ measurements were performed using the inversion recovery pulse sequence; spin–spin *T*_2_ measurements were performed using the Carr–Purcell–Meiboom–Gill (CPMG) pulse sequence^97,98^ with echo spacings of *τ* = 2 ms for most solutions. For ^19^F measurements of Mn^2+^-containing solutions, echo spacings of 1 ms were used, due to faster relaxation. NMR tubes were filled in an argon glovebox and sealed with J-Young valves.

#### Coordination to lithium salts

The following salts were used to assess transition metal behaviour at the SEI: LiOH (Alfa Aesar, 99.995%), LiF (Sigma Aldrich, 99.995%), Li_3_PO_4_ (Sigma Aldrich), Li_2_CO_3_ (Alfa Aesar, 99.998%), Li_2_O (Alfa Aesar, 99.5%), Li_2_O_2_ (Acros Organics, 95%), LiOMe (Sigma Aldrich, 98%), LiOEt (Sigma Aldrich, 95%), Li_2_C_2_O_4_ (Alfa Aesar, 99+%), LiOCHO (Alfa Aesar, 98%), LiOAc (Sigma Aldrich, 99.95%), Li(acac) (Sigma Aldrich, 97%). All salts were dried under vacuum at 100 °C before use. In plastic vials, 1 mL of 3 : 7 EC : EMC (v/v)—diamagnetic or containing 1 mM Mn(TFSI)_2_ or 1 mM Ni(TFSI)_2_—was added to 20 mg of each salt (10 mg for LiOMe, LiOEt, and Li_2_O_2_; 40 mg for LiF). Solutions were shaken twice per day; after two days, solutions were removed from the argon atmosphere and 150 μL of solution was pipetted off the top and added to 2.5% aqueous nitric acid. Samples were analysed for Li, Mn, and Ni concentration *via* ICP-OES.

### ICP-OES analysis

ICP-OES measurements were performed on an iCAP 7400 Duo ICP-OES Analyzer in axial mode (Thermo Fisher Scientific). Calibration points were measured with multi-element standard solutions at concentrations of 0.005–20.0 ppm.

## Results and discussion

### EPR spectroscopy

The following sections contain results from pulsed EPR spectroscopy of frozen electrolytes performed at a temperature of 20 K. Measurements were optimised for detecting ligand hyperfine couplings of Mn(ii) complexes, which exhibit sufficiently long electronic relaxation times for this technique.

### Field-swept EPR spectra

**Fig. 1:**
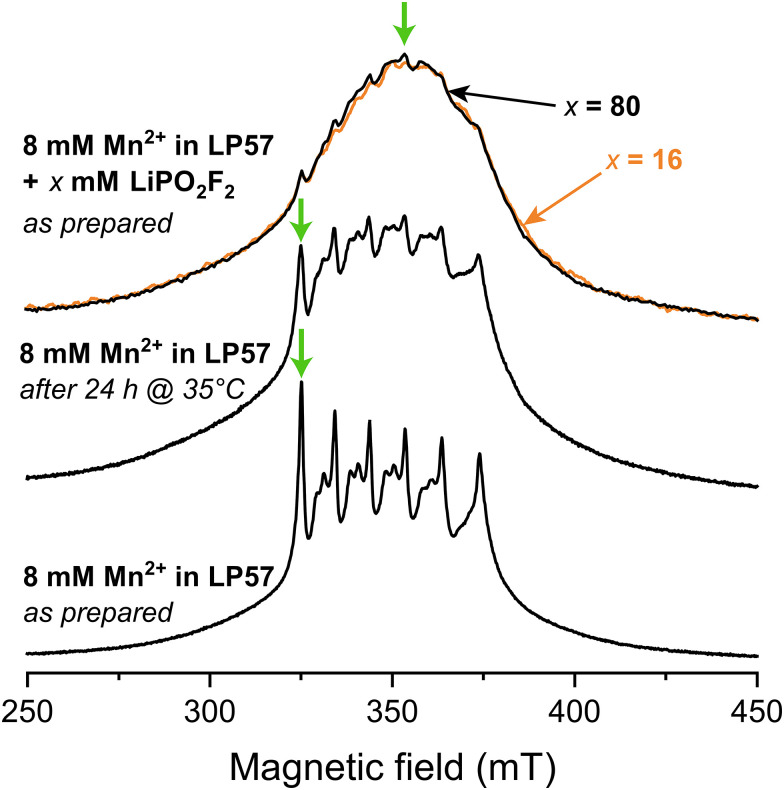
Field-swept echo-detected X-band pulsed EPR spectra, recorded at 20 K. Frozen samples of 8 mM Mn(TFSI)_2_ dissolved in 1 M LiPF_6_ in 3 : 7 EC : EMC (LP57) are investigated freshly prepared (bottom), after heating at 35 °C (middle), and with 16 mM (orange) or 80 mM (black) LiPO_2_F_2_ added (top). Orange and black arrows (top) point at the partially overlapping spectra for samples with 16 and 80 mM LiPO_2_F_2_ addition, respectively. Green arrows show the magnetic fields used for the hyperfine spectroscopy experiments (Fig. 2).

Fig. 1 shows X-band Hahn-echo-detected pulsed EPR spectra of frozen 1 M LiPF_6_ in 3 : 7 EC : EMC (LP57) electrolyte containing 8 mM Mn^2+^. The freshly prepared sample (lower spectrum) exhibits six central transition resonances corresponding to hyperfine coupling to the six nuclear spin states of the *I* = 5/2 nucleus ^55^Mn. The additional features associated with each of these peaks arise from a rhombic zero-field splitting interaction, which characterises the symmetry of the complex. This spectrum was assigned in our previous work to a sixfold coordinated Mn(ii) complex coordinated by the oxygen atoms of the carbonyl functionalities from mainly EC, and minorly EMC, with PF_6_^−^ located in the second solvation shell.^11^ When the identical sample is stored at 35 °C for 24 h, representing a mild heating protocol, the distinct six central transitions are less pronounced and the very broad outer transitions at around 300 and 400 mT are seen more clearly (middle spectrum). An analysis of the degraded electrolyte using ^19^F solution NMR revealed the presence of PO_2_F_2_^−^ and/or HPO_2_F_2_ species at a concentration of approximately 9 mM. To explore the effect of the PO_2_F_2_^−^ anion further, frozen electrolytes with LiPO_2_F_2_ salt added in a Mn^2+^ : PO_2_F_2_^−^ ratio of either 1 : 2 or 1 : 10 (corresponding to 16 and 80 mM LiPO_2_F_2_ solutions) were prepared. These now exhibit almost featureless pulsed EPR spectra, where the sharp central transitions are barely visible. Despite the different Mn^2+^ : PO_2_F_2_^−^ ratios, both samples give rise to almost identical spectra.

The increasing intensity away from the sharp central transitions and line broadening observed for the mildly heated and LiPO_2_F_2_-containing samples indicate contributions from complexes exhibiting either a large zero-field splitting, a large distribution of zero-field splitting constants, and/or strain effects.^99–101^ To estimate the relative sizes of these interactions, the zero-field splitting interactions of sixfold coordinated Mn(ii) complexes containing carbonate and fluorophosphate ligands in the first coordination shells were investigated by DFT. The zero-field splitting magnitude follows the series: carbonate (6 EC) < 6 HPO_2_F_2_/6 PO_2_F_2_^−^ ≪ mixed PO_2_F_2_^−^/HPO_2_F_2_ ≈ mixed carbonate/PO_2_F_2_^−^ ligands (SI, Fig. S1). Sixfold PO_2_F_2_^−^ coordination is, however, considered unlikely due to an overall complex charge of −4. HPO_2_F_2_ was investigated because a trace amount of water may react with LiPF_6_ under mildly elevated temperature to form HPO_2_F_2_, a species which is only weakly acidic.^102^ Although the calculated absolute values of zero-field splitting constants are often unreliable,^101^ the increase in zero field splitting for complexes containing PO_2_F_2_^−^/HPO_2_F_2_ in the Mn^2+^ coordination shell could explain the increase in broadening from the pristine sample to the mild heat-treated and LiPO_2_F_2_-additive samples. To further investigate the coordination to Mn^2+^, hyperfine-targeted pulsed EPR measurements were applied to investigate the hyperfine interactions with the various NMR active nuclei.

### X-band hyperfine sublevel correlation (HYSCORE) spectroscopy

**Fig. 2:**
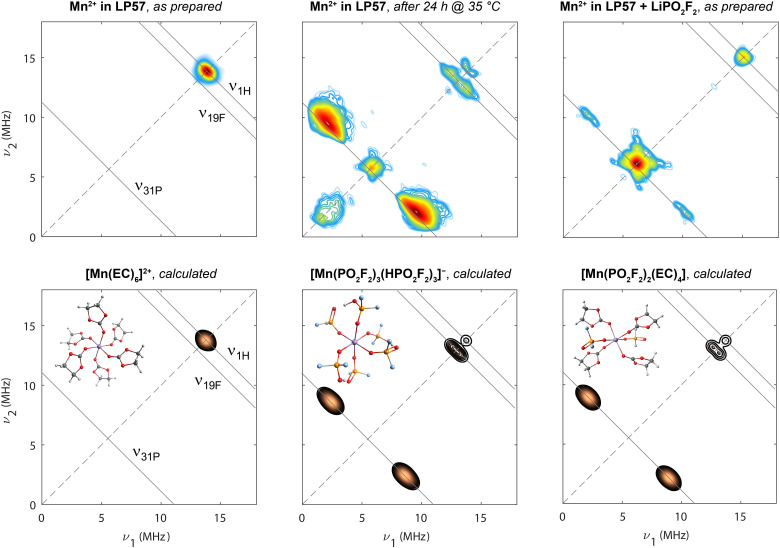
Experimental (upper row) and calculated (lower row) HYSCORE spectra at 9.8 GHz (X-band; 20 K). Spectra from samples containing 8 mM Mn(TFSI)_2_ dissolved in 1 M LiPF_6_ in 3 : 7 EC : EMC (left), with mild heat-treatment at 35 °C for 24 h (centre), and addition of 80 mM LiPO_2_F_2_ (right) are shown. Experiments were conducted at the magnetic field positions indicated by green arrows in Fig. 1. The diagonal is shown with a dashed line. Anti-diagonal (solid) lines are shown at relevant nuclear Larmor frequencies. Calculations are shown for three clusters, [Mn(EC)_6_]^2+^, [Mn(PO_2_F_2_)_3_(HPO_2_F_2_)_3_]^−^ and [Mn(PO_2_F_2_)_2_(EC)_4_].

The above-introduced samples of Mn(ii) in 1 M LiPF_6_/3 : 7 EC : EMC (LP57), either freshly prepared, mildly heat-treated, or with added LiPO_2_F_2_, were first analysed by HYSCORE spectroscopy (Fig. 2, upper row). This four-pulse, two-dimensional EPR experiment is performed at a constant magnetic field (*i.e.*, at one particular position in the EPR spectrum, as marked in Fig. 1 with green arrows) and detects coupling to magnetic nuclei in the solvation sphere(s) of the Mn(ii) ion, with the nuclear transitions *ν*_*α*,*β*_ (for *S* = 1/2) being observed, to first order, at frequencies*ν*_*α*,*β*_ = *ν*_I_ ± *A*/2 if |*A*/2| < *ν*_I_centred at the nuclear Larmor frequency *ν*_I_ with hyperfine coupling constant *A*. Possible nuclei are ^1^H from EC and EMC as well as ^6,7^Li, ^19^F, and ^31^P from the electrolyte salt LiPF_6_, while ^13^C and ^17^O are not sensitive enough due to their low natural abundance. Nuclei in impurities or degradation products may also need to be considered if these species become ligands. The time-domain detection allows these magnetic nuclei, with their respective hyperfine coupling, to be probed simultaneously, and the two-dimensionality increases resolution since, first, nuclear frequencies are spread along the diagonal and, second, the splitting between peaks on the anti-diagonal corresponds to the hyperfine coupling constant *A*. The spectrum of the pristine sample solely exhibits an intense signal on the diagonal at the nuclear Larmor frequency *ν*_^1^H_, indicating weakly coupled and thus remote ^1^H nuclei. This is in line with proposed carbonate coordination with Mn–H distances of ≥ 5.5 Å.^11^

After mild heating of the Mn(ii)-containing sample, the resonance at *ν*_^1^H_ splits, indicating a larger ^1^H hyperfine interaction, and new resonances centred at *ν*_^31^P_ and *ν*_^19^F_ appear, indicating a change of the Mn^2+^ coordination shell. The most intense signals cluster around the ^31^P anti-diagonal with hyperfine coupling constants of around *A* = 5.5–10 MHz. While the intensity of HYSCORE signals is determined by the number of coupled nuclei, the coordination number is difficult to directly determine, as the signal intensifies when *A*/2 approaches *ν*_I_ (*ν*_^31^P_ = 5.6 MHz) and is also affected by experimental parameters. The separate *ν*_^31^P_ signal on the diagonal indicates additional, more remote ^31^P-containing species in outer solvation spheres with weaker but non-zero hyperfine coupling constants. Coupling parameters of *A*(^19^F) = 1.3 MHz and *A*(^1^H) = 0.6 MHz are extracted from the observed maximum antidiagonal intensity at the respective Larmor frequencies. The HYSCORE signals exhibit a specific curvature, which is pronounced for ^1^H and to some degree for ^31^P and ^19^F: generally, the further away resonances are from the diagonal, the more these resonances move away from the anti-diagonal toward higher frequencies, as discussed in more detail in ref. 103. This line shape results from a continuous variation of hyperfine tensor values due to a conformational distribution rather than exactly defined atomic positions in the frozen state,^91,103,104^ which is also consistent with the observed line broadening in Fig. 1. The shift away from the nuclear Larmor frequency towards higher frequency directly on the diagonal, as best seen for ^31^P but also to some degree for ^1^H, is indicative of the dipolar contribution to the hyperfine interaction.^105^ The signal at low *ν*_1,2_ results from imperfect microwave pulses and limited excitation bandwidth.

For the sample with added LiPO_2_F_2_, only signals due to coupling to ^31^P and ^1^H are resolved. A doublet on the *ν*_^31^P_ antidiagonal from strongly coupled ^31^P spins is detected at identical frequencies as observed for the mildly heated sample; however, it is far less intense. In contrast, the intensity maximum is found on the diagonal, the signal arising from a large number of weakly interacting and thus more remote ^31^P spins. The ^1^H signal is also located on the diagonal and is not as clearly split as for the mildly heated sample, again indicating more remote ^1^H nuclei.

DFT calculations of *A*_*x*,*y*,*z*_ and subsequent spectra simulation (Fig. 2, lower row) allow a variety of structural models to be compared to the experimental data. For the pristine sample, a simulated HYSCORE spectrum of a Mn(ii) complex sixfold coordinated by EC provides a good match to the experiment. The broadening of the diagonal peak, seen in the spectral simulations and assigned to weak coupling to nearby ^1^H spins, arises from both the broadening due to *T*_2e_ relaxation, with the values used in this simulation specified in the Methods section, and the slightly varying hyperfine tensors for all 24 involved weakly coupled ^1^H nuclei. In the experiment, nuclei from outer solvation shells and conformational differences between complexes also contribute to the broadening, which are both disregarded in all simulations. Mn(ii) complexes with sixfold coordination containing either both fluorophosphate and EC ligands, or both HPO_2_F_2_ and EC ligands resemble the experimental spectrum of the mildly heat-treated sample, consistent with complexes proposed above. Other structure models, including bidentate oxygen coordination of PO_2_F_2_^−^, or PF_6_^−^ coordination as a source for ^31^P and ^19^F couplings, are precluded, since they lead to hyperfine coupling values or simulated HYSCORE features that are not consistent with the experimental spectra as, for example, the coupling to the ^31^P spins is too weak (Fig. S2). The underlying ratio of protic HPO_2_F_2_ to aprotic PO_2_F_2_^−^ is not extractable, because the effect of protonation on the simulated spectrum is minor (Fig. S2). The weaker peak seen on the diagonal near the ^31^P frequency in the experimental spectrum is assigned to P-containing complexes that are in more distant (outer sphere) coordination shells: all P-containing ligands—even PF_6_^−^—in the first coordination shell give rise to pronounced ^31^P hyperfine coupling.

DFT seems to underestimate hyperfine coupling strengths, particularly for ^19^F and ^1^H, as also observed for predicted ^55^Mn coupling tensors (Table S1). As discussed previously,^106,107^ d^5^ systems still prove particularly challenging for DFT due to the large electron–electron exchange and correlation interactions, also manifested in DFT functional sensitivity (Fig. S1 and S2). Apart from DFT errors, the optimised lowest-energy structures also likely deviate significantly from the experimental situation. The proposed Mn(ii) fluorophosphate structures, and in particular the lowest-energy location of the proton in the HPO_2_F_2_ ligands, depend on the HPO_2_F_2_ : PO_2_F_2_^−^ ratio (Fig. S3). Whereas in [Mn(HPO_2_F_2_)_6_]^2+^, all –OH groups face away from the center of the complex, with Mn–H distances of 5.0–5.5 Å, inter-ligand hydrogen bonds are formed in [Mn(PO_2_F_2_)_3_(HPO_2_F_2_)_3_]^−^, resulting in [–Mn–O–P–O–H–O–P–O–] rings with shorter Mn–H distances of ∼3.9 Å. Hydrogen bonding *via* smaller rings, *e.g.* [–Mn–O–P–O–H–O–], is also conceivable. Additionally, outer solvation shells might affect bond lengths, angles, and dihedrals, which have a large effect on all hyperfine coupling tensors of the complex,^91,103^ which might explain deviations between experimental and simulated spectra.

Considering the sample with LiPO_2_F_2_, the experimental spectrum is noticeably different from the mildly heated electrolyte sample's HYSCORE spectrum and does not reflect pure inner sphere fluorophosphate coordination. Assuming a lack of protic HPO_2_F_2_ in this solution and charge neutrality, the first structural model considered was a [Mn(PO_2_F_2_)_2_(EC)_4_] complex. The reduced symmetry and therefore larger zero-field splitting (Fig. S1) could explain the broad field-swept EPR spectrum (Fig. 1). Although the intensity contribution of ^1^H resonances increases relative to complexes with no EC molecules, agreement with the experimental HYSCORE spectrum is still poor. Nonetheless, this and related complexes are still likely responsible for the weak signals along the ^31^P anti-diagonal, with associated couplings to ^19^F and ^1^H too weak to be resolved. The intense ^31^P resonance near the diagonal indicates the presence of a superimposed second complex type, the stronger ^1^H diagonal peak than seen for the degraded electrolyte suggesting that more EC ligands are present in the Mn^2+^ coordination shell. In contrast to the mildly heated sample, fluorophosphate is present as unprotonated PO_2_F_2_^−^. A preference of PO_2_F_2_^−^ for Li^+^ might hinder Mn^2+^ coordination, placing it in outer solvation shells of Mn^2+^ resulting in an intense ^31^P signal on the diagonal, indicating weaker hyperfine interactions. The underlying Mn(ii) carbonate complex would account for the ^1^H diagonal signal. Additional experiments show that the relative ratio of strongly to weakly coupled ^31^P increases with the LiPO_2_F_2_ : Mn^2+^ ratio (Fig. S4, ratio ranging from 2 : 1 to 10 : 1). This indicates that PO_2_F_2_^−^ is more likely to move from an outer to the inner solvation shell with increasing PO_2_F_2_^−^ concentration, following the law of mass action.

Another possible explanation of the HYSCORE spectra involves the formation of Mn(ii) complexes containing side products as ligands. A number of (oligo-)organo-(fluoro)phosphates can form through PO_2_F_2_^−^ reaction with EC or EMC,^108,109^ which could bind to Mn^2+^*via* carbonate fragments that are chemically bonded to phosphate(s), accounting for the HYSCORE ^31^P and ^1^H signals. However, we consider such degradation products unlikely because significant amounts only form above 40 °C,^110^ and the ^19^F NMR spectra of these samples showed that the dominant degradation product is PO_2_F_2_^−^/HPO_2_F_2_.

### Q-band ENDOR

**Fig. 3:**
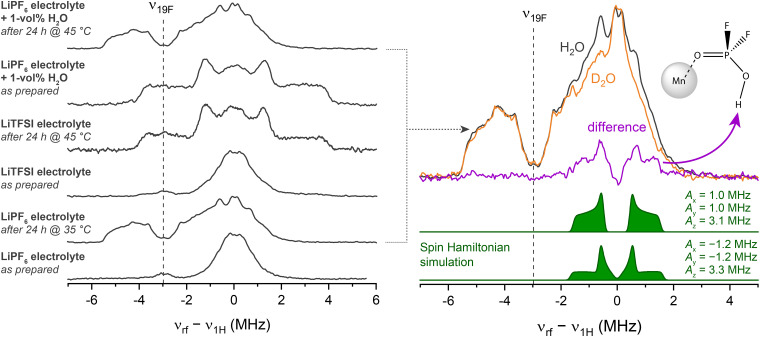
Davies-type electron nuclear double resonance (ENDOR) spectra recorded at 34 GHz (Q-band) of a series of samples containing 8 mM Mn^2+^ in a 3 : 7 EC : EMC solvent with 1 M Li salt (LiPF_6_ and LiTFSI) as indicated (left). The radiofrequency axis is shifted to yield the ^1^H Larmor frequency at 0 MHz. Right: ENDOR spectra of the LiPF_6_-containing electrolyte with either 1 vol% H_2_O (black) or D_2_O (orange). Spin Hamiltonian fits to these spectra (green) were adjusted to reproduce the difference spectrum (purple). The complex shown, Mn(HPO_2_F_2_), was used to simulate the spectra, with simulations for two different axial hyperfine tensors being shown.

To support the assumption that trace moisture is responsible for HPO_2_F_2_ formation in the mildly heated sample, Davies-type electron nuclear double resonance (ENDOR) spectroscopy at Q-band was performed. In an ENDOR experiment, radiofrequency irradiation *ν*_rf_ drives nuclear transitions and, thus, modifies the evolution of magnetization under the (isotropic and anisotropic) hyperfine interaction, altering the electron spin echo intensity. This experiment often allows the principal components of the hyperfine tensor *A*_*x*,*y*,*z*_ to be extracted, unlike in a HYSCORE experiment, where the modulation amplitude tends to vanish for orientations aligned with the principal axes.^111^ Here, the method is used to compare the ^19^F–Mn^2+^ and ^1^H–Mn^2+^ hyperfine interactions in multiple electrolytes, including electrolytes containing either LiPF_6_ or LiTFSI salts, before and after heating and with added water (Fig. 3). Their corresponding 1D field-swept EPR spectra are displayed in Fig. S5. The pristine LiPF_6_ electrolyte sample exhibits an intense ENDOR signal at around *ν*_^1^H_ (50.45 MHz) assigned to carbonates, and a low-intensity signal around *ν*_^19^F_ assigned to PF_6_^−^ in outer solvation shells (Fig. 3, left panel, bottom spectrum).^11^ After mild heating (24 h at 35 °C), the hyperfine coupling to both ^19^F and ^1^H is larger; the signals centred around *ν*_^19^F_ become more intense, with the more distinct features indicating better defined complexes. Since fluorophosphates result from LiPF_6_ decomposition, pristine and heated (24 h at 45 °C) samples were also prepared in a LiTFSI-based, but otherwise identical, electrolyte for comparison. The spectrum of the pristine LiTFSI electrolyte resembles that of the LiPF_6_ electrolyte. After heating, however, a very different ligand environment is detected, with couplings (predominantly from ^1^H) that are in very good agreement with the spectral fingerprint of [Mn(OH_2_)_6_]^2+^.^112,113^ Water has clearly been formed, either as part of an electrolyte degradation reaction or because it has entered into the NMR tube during the heating step. When 1 vol% (0.56 M) H_2_O was intentionally added to the LiPF_6_ electrolyte pristine sample, a very similar spectrum to that seen for the LiTFSI heated sample, *i.e.*, [Mn(OH_2_)_6_]^2+^, is observed. However, after heating this sample for 24 h at 45 °C, the spectrum (Fig. 3, top right) is essentially identical to that of the mildly heated sample without water addition, indicating that fluorophosphate has formed, and suggesting that fluorophosphate coordination is preferred over H_2_O coordination.

The ENDOR spectrum of this mildly heated (wet) LiPF_6_ electrolyte sample contains multiple overlapping hyperfine coupling patterns, possibly from more than one species. To reduce complexity, D_2_O instead of H_2_O was added to the solution so as to form DPO_2_F_2_*via*

1 LiPF_6_ + 2D_2_O → DPO_2_F_2_ + 3DF + LiF

Contributions from deuterium are shifted away to a different frequency regime in the ENDOR spectrum because *ν*_2_D__ is 6.5 times smaller than *ν*_^1^H_.^70^ The difference between the spectra of the H_2_O and D_2_O-containing electrolytes reduces to a simple ^1^H coupling pattern, which exhibits a distinctly different spectral fingerprint from that of water ligands (Fig. 3, right panel). The coupling pattern is characteristic of an axially symmetric hyperfine coupling tensor (*A*_*x*_ = *A*_*y*_ ≠ *A*_*z*_), which indicates a single or multiple, but magnetically very similar, ^1^H position(s). Assuming a single contributing ^1^H nucleus, two sets of hyperfine coupling constants are conceivable due to sign ambiguities: (1.0, 1.0, 3.1) or (−1.2, −1.2, 3.3) in MHz. The anisotropic part can be directly derived from these principal components, and used to estimate the electron–nuclear distance by assuming point-dipoles. This approximation is inaccurate for a transition metal complex with delocalised spin density (Fig. S3), but may serve as method for extracting a rough orientation of the complex. The two sets of hyperfine coupling constants imply Mn–H distances of 4.8 and 3.7 Å, respectively, lying in the range of DFT optimised distances of 3.9–5.5 Å. The residual resonances centred around *ν*_^1^H_ are from non-water sources and correspond to comparably low coupling strengths with *A* ≈ 0.2 MHz; these are assigned to outer solvation shells and/or remaining carbonate ligands in the first solvation shell. For resonances centred at *ν*_^19^F_, three characteristic features can be extracted, which are seen more clearly at lower frequencies compared to *ν*_^19^F_ since there is no overlap with the resonances associated with ^1^H. The lowest-frequency rising edge at *ν*_rf_ − *ν*_^1^H_ ≈ −5.4 corresponds to *A* ≈ 4.7 MHz, the local maximum to 2.8 MHz, and the falling edge to 1.3 MHz. The unknown number of contributing nuclei and the unknown tensor symmetry impedes further analysis, but do indicate that an inner sphere complex, with large ^19^F hyperfine interactions, is present. Additional EPR studies using deuterated additives were performed to explore their ability to displace carbonate/fluorophosphate ligands from the Mn^2+^ solvation shell (Fig. S6). Results indicate that displacement from the first coordination sphere is attained with CD_3_OD, partially with d_6_-DMSO, and to some degree with CD_3_CN.

### NMR spectroscopy

The paramagnetic relaxation theory underpinning this section can be found in our previous work,^11^ and in many other reviews;^114^ a condensed version is also presented in the SI.

### NMR relaxometry of degraded electrolyte solutions

**Fig. 4:**
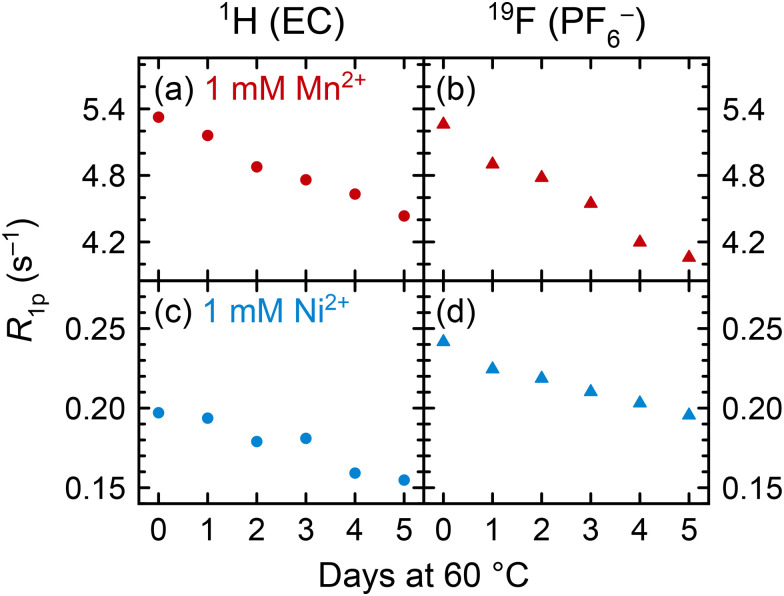
(a) and (c) ^1^H EC (circles) and (b) and (d) ^19^F PF_6_^−^ (triangles) longitudinal paramagnetic relaxation rates (*R*_1p_) of Mn^2+^- or Ni^2+^-containing electrolyte solutions across five days of heating at 60 °C. Electrolyte solutions comprised 1 M LiPF_6_ in 3 : 7 EC : EMC (v/v) with 1 mM Mn(TFSI)_2_ (red) or Ni(TFSI)_2_ (blue). Solutions were cooled to ambient temperature (∼25 °C) before relaxation rates were measured.

To understand how coordination may change between pristine and degraded electrolyte solutions, pristine solutions were heated at 60 °C for five days and the spin lattice *R*_1_ (= 1/*T*_1_) relaxation rates were measured daily. Relaxation rates of 1 M LiPF_6_ in 3 : 7 EC : EMC electrolyte solutions containing either 1 mM Mn(TFSI)_2_ or 1 mM Ni(TFSI)_2_ are presented in Fig. 4 as paramagnetic relaxation rates (*R*_1p_). Values were calculated by subtracting the relaxation rates *R*_1d_ of pristine diamagnetic electrolyte solutions from the measured relaxation rates, *R*_1p_ = *R*_1_ − *R*_1d_,^62^ where *R*_1d_ for 1 M LiPF_6_ in 3 : 7 EC : EMC v/v is 0.6307 ± 0.0002 s^−1^ for ^1^H of EC and 0.352 ± 0.003 s^−1^ for ^19^F of PF_6_^−^, as determined in our previous work.^115^ This method assumes that the *R*_1d_ relaxation rates of pristine solutions remain constant over a period of heating, or with the addition of a small amount of contaminant. We suggest that minor compositional changes to the electrolyte should not significantly affect bulk properties such as viscosity, and that the concentration of degradation species is small relative to the other solution components: 1 M LiPF_6_, 4.5 M EC, and 6.8 M EMC.

In both Mn^2+^- and Ni^2+^-containing electrolyte solutions, the ^1^H (EC) and ^19^F (PF_6_^−^) *R*_1p_ values show a clear decrease across the heating period. With no substantial change in the total concentration of solution components, the decrease in *R*_1p_ values indicates a decrease in the number of EC or PF_6_^−^ molecules in the transition metal solvation shells. This suggests that the transition metal ions must be preferentially coordinating to a new component in the degraded electrolyte. (Electrolyte degradation species were not observable in these samples owing to both the paramagnetic ions and the use of a solid-state spectrometer for these measurements; however, later NMR experiments with heated diamagnetic electrolyte showed PO_2_F_2_^−^ and HF as the major degradation species (Fig. 7), which is also consistent with analysis of heated EPR samples.) While the lifetimes of the different Mn^2+^/Ni^2+^ complexes may change, affecting NMR relaxation times,^11^ the EPR results in the previous section clearly indicate changes in the nature of the complexes present in the degraded electrolyte, consistent with the NMR results shown here.

### Transition metal coordination to PO_2_F_2_^−^

**Fig. 5:**
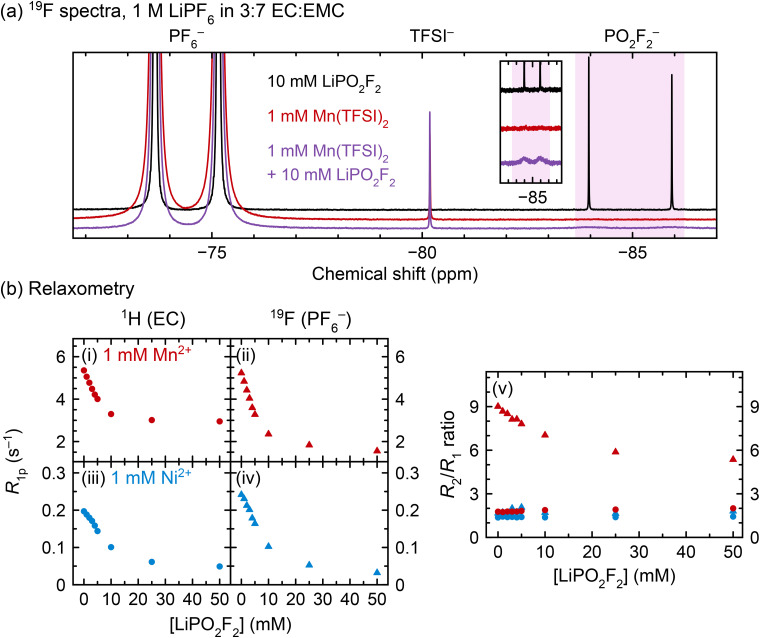
(a) ^19^F spectra and (b) ^1^H EC and ^19^F PF_6_^−^ relaxation data of pristine electrolyte solutions to which LiPO_2_F_2_ was added. ^19^F spectra are shown for solutions containing LiPO_2_F_2_, Mn^2+^, or both; inset shows enlargement of the PO_2_F_2_^−^ resonance. Longitudinal paramagnetic relaxation rates (*R*_1p_) and *R*_2_/*R*_1_ ratios are shown for the ^1^H EC (circles) and ^19^F PF_6_^−^ resonances (triangles), in solutions containing Mn^2+^ (red) or Ni^2+^ (blue). Electrolyte solutions comprised 1 M LiPF_6_ in 3 : 7 EC : EMC (v/v).

To isolate the effect of PO_2_F_2_^−^ on transition metal coordination, LiPO_2_F_2_ was directly added to pristine electrolyte solutions. Fig. 5a shows NMR spectra for 1 M LiPF_6_ in 3 : 7 EC : EMC (v/v) containing added LiPO_2_F_2_, Mn^2+^, or both; Fig. 5b shows relaxation measurements for solutions containing 1 mM Mn^2+^ or Ni^2+^ and 0–50 mM LiPO_2_F_2_.

As expected, the ^19^F NMR spectrum of the diamagnetic solution containing LiPO_2_F_2_ (Fig. 5a, black line) shows narrow doublets for both PF_6_^−^ and PO_2_F_2_^−^, while the solution that contains Mn^2+^ but not LiPO_2_F_2_ (red line) shows a broadened PF_6_^−^ doublet only. However, in the solution containing both Mn^2+^ and LiPO_2_F_2_ (purple line), the PF_6_^−^ doublet is much narrower than its counterpart in the Mn^2+^-only solution; additionally, the PO_2_F_2_^−^ doublet is severely broadened relative to the PO_2_F_2_^−^ doublet in the diamagnetic solution, indicating faster relaxation. The same behaviour can be seen in Fig. 5b, panel (ii), where the ^19^F PF_6_^−^*R*_1p_ values decrease in Mn^2+^-containing solutions as LiPO_2_F_2_ is added.

In Fig. 5b, panels (i–iv), the ^1^H (EC) and ^19^F (PF_6_^−^) *R*_1p_ values decrease with increasing LiPO_2_F_2_ concentration. ^1^H EMC relaxation rates also decrease (not shown). The diamagnetic relaxation rates should not be affected in these samples as the solution viscosity increase due to the addition of 0–50 mM LiPO_2_F_2_ is likely minor. Regardless, a viscosity increase would lead to an increase rather than a decrease in relaxation rates, as the solutions are in the fast motion regime.^11^ The decrease in *R*_1p_ values is consistent with the results in Fig. 4, where EC and PF_6_^−^ relaxation rates slowed as PO_2_F_2_^−^ and other fluorophosphates were generated *in situ* by heating. The decrease in *R*_1p_ values in Fig. 5b begins to level off at ∼10–25 mM LiPO_2_F_2_ addition, indicating a very strong competition for Ni^2+^/Mn^2+^–PO_2_F_2_^−^ complexation over Ni^2+^/Mn^2+^–PF_6_^−^ interactions/complexes. The larger decrease in ^19^F *R*_1p_ rates over the ^1^H values suggests that EC is still involved in binding to Ni^2+^/Mn^2+^ even in the presence of PO_2_F_2_^−^, which is consistent with the EPR results.

Fig. 5b, panel (v) shows the *R*_2_/*R*_1_ ratios for the same solutions studied in panels (i)–(iv). The only instance where the *R*_2_/*R*_1_ ratio exceeds 2 is the ^19^F PF_6_^−^ relaxation in Mn^2+^-containing solutions (red triangles), where the *R*_2_/*R*_1_ ratio decreases from 9.0 to 5.4 as 50 mM LiPO_2_F_2_ is added. This indicates that a different relaxation mechanism is driving the *R*_2_ rates in this system. As discussed in our previous paper,^11^ and explored further in the SI, one potential source of an *R*_2_/*R*_1_ ratio greater than one is a contribution of the contact (through-bond) relaxation mechanism to *R*_2_, while the major source of *R*_1_ relaxation originates from a dipolar (though-space) interaction. Thus, an *R*_2_/*R*_1_ ratio of ≫1 could be ascribed to a short-lived inner sphere complex. Since the hyperfine interaction for a Mn^2+^–PF_6_^−^ inner sphere complex should remain the same with added LiPO_2_F_2_, the convergence of *R*_2_ towards *R*_1_ could then be ascribed to a decrease in the relative fraction of inner sphere *vs.* outer sphere Mn^2+^–PF_6_^−^ complexes. However, the HYSCORE EPR spectrum shows no evidence of an inner sphere complex—*i.e.*, no strong ^19^F/^31^P–Mn^2+^ hyperfine interaction is seen, while the ENDOR spectrum of the same sample reveals only a weak interaction likely due to an anisotropic (dipolar, through-space) interaction, indicating the presence of some PF_6_^−^ anions in the outer coordination shells in the frozen electrolytes. Thus, the time that a PF_6_^−^ anion spends on average in an inner sphere complex must be extremely short, so that it is not captured in the frozen solution. It is also possible that a second relaxation mechanism involving a change in the dynamics of exchange in and out of the outer sphere coordination shell may contribute to some of the observed changes in the relaxation processes, but to test this proposal is beyond the scope of this work.

While it could be argued that the PO_2_F_2_^−^ addition slowed the ^1^H and ^19^F relaxation rates by causing Mn^2+^ precipitation, it has been shown that LiPO_2_F_2_ does not cause transition metal precipitation in battery electrolytes.^32^ Two experiments in this work also suggest that precipitation does not occur: (i) when a solution of 1 mM Mn^2+^ in 3 : 7 EC : EMC was saturated with LiPO_2_F_2_, ICP-OES measurement revealed that the Mn concentration in solution was unchanged; (ii) the NMR spectra in Fig. 5a show that when 10 mM LiPO_2_F_2_ is added to a solution of 1 M LiPF_6_ in 3 : 7 EC : EMC + 1 mM Mn^2+^, the PO_2_F_2_^−^ resonance becomes extremely broad, but does not disappear, indicating that it is interacting with Mn^2+^ but is still present in solution. Notably, the result of transition metal coordination to PO_2_F_2_^−^ in solution, shown by both EPR and NMR, may also apply to other cell chemistries, as PF_6_^−^ salts are used in beyond-lithium systems, including sodium-ion, potassium-ion, and calcium-ion cells.^116^

### Transition metal coordination to other model degradation species

**Fig. 6:**
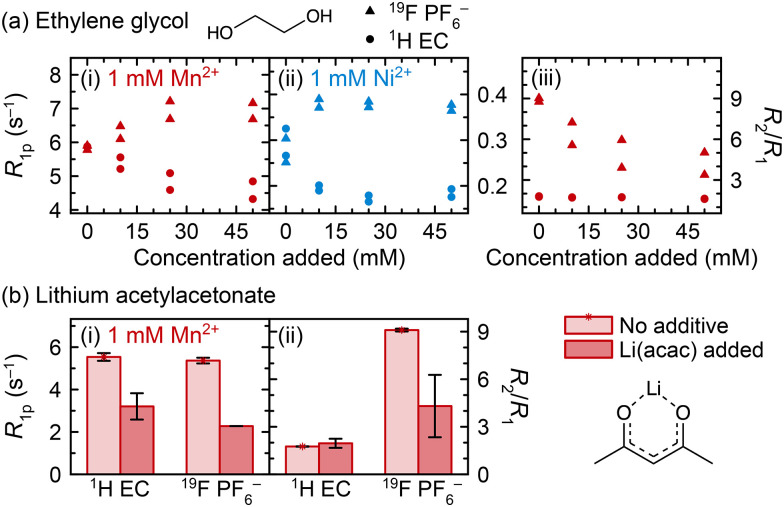
Relaxation behaviour upon either (a) ethylene glycol or (b) Li(acac) addition to paramagnetic electrolyte solutions. *R*_1p_ values and *R*_2_/*R*_1_ ratios are shown for ^19^F PF_6_^−^ resonances (triangles) and ^1^H EC resonances (circles) in electrolyte solutions containing 1 mM Mn(TFSI)_2_ (red) or Ni(TFSI)_2_ (blue). The electrolyte solution comprises 1 M LiPF_6_ in 3 : 7 EC : EMC (v/v).

We now explore Mn^2+^ and Ni^2+^ coordination to two other degradation species, ethylene glycol (EG) and acetylacetonate (acac^−^) (Fig. 6). The solubility of Li(acac) in the electrolyte solution was found to be very small, so data are presented here for solutions that are saturated with Li(acac), roughly estimated to occur at ∼5 mM Li(acac). Fig. 6a shows that after ethylene glycol addition to Mn^2+^- or Ni^2+^-containing solution, the ^1^H (EC) *R*_1p_ values decrease, suggesting that ethylene glycol replaces (at least some) EC in the Mn^2+^ and Ni^2+^ solvation shells. Interestingly, instead of the ^19^F PF_6_^−^*R*_1p_ values decreasing, as occurs upon LiPO_2_F_2_ addition, the *R*_1p_ values increase. This suggests that PF_6_^−^ is in closer proximity to Mn^2+^ or Ni^2+^ when ethylene glycol is present. It is not likely that the fraction of coordinated PF_6_^−^ increases, because EC coordination is preferred over PF_6_^−^ coordination and so the displacement of EC by an even more preferred coordination agent should not then facilitate additional PF_6_^−^ binding.

Concurrently, the ^1^H (EC) *R*_2_/*R*_1_ ratio remains constant and the ^19^F (PF_6_^−^) *R*_2_/*R*_1_ ratio decreases in Mn^2+^-containing solutions. It is noted here that the absolute value of *R*_2_ decreases, not just the *R*_2_/*R*_1_ ratio—if the *R*_2_/*R*_1_ decrease were caused by *R*_2_ remaining constant as *R*_1_ increased, then *R*_2_ would only decrease by 11–18%, not 43–63% as is observed. Together, the longitudinal and transverse relaxation behaviour is consistent with the interpretation of an inner solvation shell mostly composed of EC, which is displaced by ethylene glycol: the smaller size of ethylene glycol compared to EC results in a smaller first solvation shell, resulting in a closer approach by PF_6_^−^ ions in the second solvation shell to Mn^2+^ or Ni^2+^, along, potentially, with different dynamics for PF_6_^−^ exchange, and an increase in *R*_1_ and decrease in *R*_2_. It is possible that EG binding reduces the albeit small probability that PF_6_^−^ forms an inner sphere complex, also reducing the *R*_2_/*R*_1_ ratio.

We have previously suggested coordination to the ethylene glycol dianion for vanadyl ions dissolved in degraded LiPF_6_ electrolyte solutions, supported by pulsed EPR measurements.^70^ We note that the experiments in Fig. 6a used neutral ethylene glycol, but it is possible that if it exists *in situ* as the dianion, then the transition metal charge would be satisfied by the anionic ethylene glycolate, and coordination to PF_6_^−^ may become significantly less favoured. The acidity of the electrolyte solution likely helps determine the favourability of transition metal coordination by determining whether potential coordinating ligands are neutral or charged.

The addition of Li(acac) to Mn^2+^-containing electrolyte (Fig. 6b) causes similar results as addition of LiPO_2_F_2_ (Fig. 5b), where the ^1^H (EC) and ^19^F (PF_6_^−^) *R*_1p_ values both decrease and the ^19^F (PF_6_^−^) *R*_2_/*R*_1_ ratio also decreases, suggesting both EC and PF_6_^−^ are displaced from the inner and outer spheres. Notably, the ICP-OES results that will be discussed later show that Li(acac) causes precipitation of ∼9% of 1 mM Mn^2+^ in 3 : 7 EC : EMC, which may have contributed to the observed decrease in *R*_1p_ values. Still, the qualitative interpretation of the NMR data here and the conclusion that acac^−^ affects the Mn^2+^ solvation shell is reasonable, because the dramatic change in the ^19^F (PF_6_^−^) *R*_2_/*R*_1_ ratio shows that the results in Fig. 6b are not only due to loss of overall Mn^2+^ in solution. Had a small amount of Mn^2+^ simply precipitated, and the overall concentration of Mn^2+^ were slightly smaller, the ^19^F (PF_6_^−^) *R*_2_/*R*_1_ ratio would not be expected to change significantly.

### Electrolyte degradation induced by dissolved transition metals

**Fig. 7:**
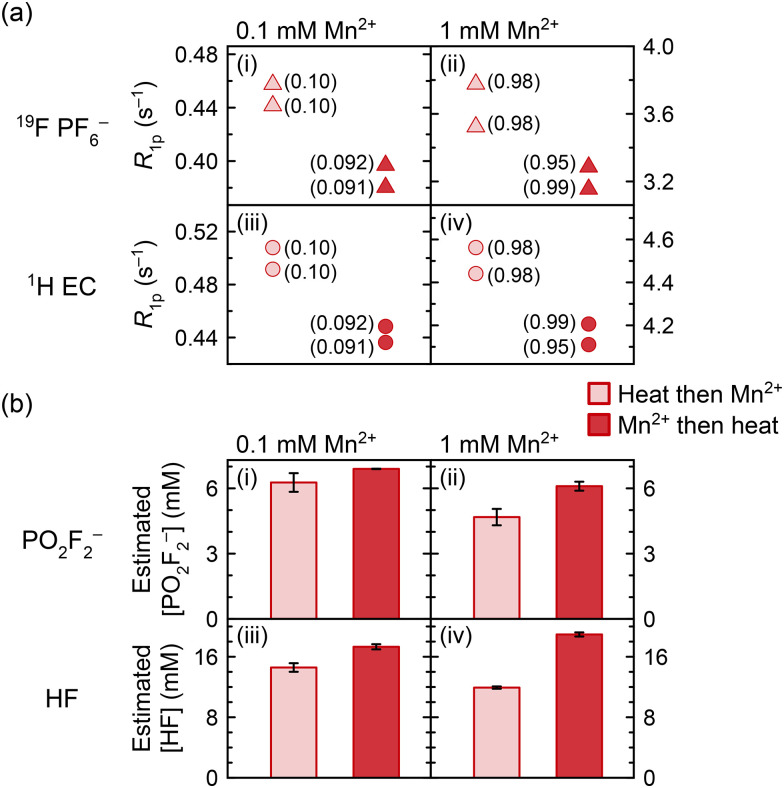
(a) ^19^F PF_6_^−^ (triangles) and ^1^H EC (circles) longitudinal paramagnetic relaxation rates (*R*_1p_) and (b) estimated concentrations of PO_2_F_2_^−^ and HF in electrolyte solutions to which Mn^2+^ was added before (red) or after heating (pink) at 60 °C for ten weeks. Values next to the relaxation data indicate the Mn concentration in mM measured by ICP-OES. PO_2_F_2_^−^ estimated concentrations were derived from integrating ^19^F spectra relative to PF_6_^−^ (assuming 1 M PF_6_^−^); HF estimated concentrated were derived from ^19^F spectra as well as from ^1^H spectra relative to EC (assuming 4.5 M EC). Electrolyte solutions comprised 1 M LiPF_6_ in 3 : 7 EC : EMC (v/v), to which 0.1 or 1.0 mM Mn(TFSI)_2_ was added.

Previous work has asserted that dissolved Mn^2+^ can itself induce PF_6_^−^ degradation.^8,14^ This is now explored in work presented in Fig. 7, which shows the ^19^F (PF_6_^−^) and ^1^H (EC) relaxation rates, as well as the measured concentrations of PO_2_F_2_^−^ and HF generated, in solutions of 0.1 mM and 1.0 mM Mn^2+^. In one group of samples, Mn^2+^ was added before heating at 60 °C for ten weeks, and in the other group, Mn^2+^ was added after heating; if the Mn^2+^ ions were inert, results should be identical. NMR of the heated diamagnetic sample prior to any Mn^2+^ addition indicated that PO_2_F_2_^−^ and HF were the major degradation species. Mn concentrations were confirmed with ICP-OES and are indicated in parentheses in Fig. 7a; we note that duplicate samples were prepared from separate stock solutions of Mn^2+^, accounting for the small variation in Mn concentrations between some duplicates.

Since the generation of fluorophosphate degradation species results in reduced PF_6_^−^ and EC relaxation rates, as the fluorophosphates compete for the Mn^2+^ ions (Fig. 4 and 5), the relaxation rates may be used as a proxy to monitor the degree of degradation in electrolyte solutions with the same Mn^2+^ concentration. In Fig. 7a, ^1^H (EC) and ^19^F (PF_6_^−^) longitudinal paramagnetic relaxation rates are slower in all samples that were heated in the presence of Mn^2+^, compared to samples where Mn^2+^ was added after heating. However, ICP-OES also showed a small but significant difference in the concentration of the nominally 0.1 mM samples, where the samples with Mn^2+^ addition before heating contained 0.1 mM Mn but the samples with Mn^2+^ addition after heating contained 0.09 mM Mn (attributed to experimental error in solution preparation). This difference in concentration is sufficient to explain most of the relaxation difference between the datasets. While small differences in Mn concentrations were also seen in the samples nominally containing 1.0 mM of Mn^2+^, the sample containing 0.99 mM Mn added pre-heating produced slower relaxation rates than both samples containing 0.98 mM Mn added post-heating. This suggests that paramagnetic samples underwent more degradation than diamagnetic samples; *i.e.*, the Mn(TFSI)_2_ addition caused an albeit small amount of additional electrolyte degradation.

The samples were then diluted 10× with d_6_-DMSO to improve the visibility of the degradation peaks, as has been discussed elsewhere.^117^ Further NMR analysis concerning the effects of deuterated solvents, including d_6_-DMSO, CD_3_CN, and MeOD, on the Mn^2+^ solvation shell is provided in the SI (Fig. S7); the most significant effects are observed with DMSO, which appears to displace EC from the solvation shell. The resulting ^19^F spectra were used to estimate the concentrations of PO_2_F_2_^−^ and HF (Fig. 7b). These are still only estimates, as the d_6_-DMSO does not completely prevent the broadening of degradation peaks; hence, comparisons are only drawn between samples containing the same Mn^2+^ concentrations, and not between the 0.1 and 1.0 mM datasets. In all cases, the PO_2_F_2_^−^ and HF concentrations are larger in the samples that were heated in the presence of Mn^2+^. It is possible that some of the degradation enhancement is due to the Mn(TFSI)_2_ addition requiring several transfers during the preparation and dilution of stock solutions, which may have caused minor contamination from trace adsorbed water on vial or pipette surfaces, despite work being carried out in an argon glovebox. That said, the degradation enhancement is larger when more Mn^2+^ is present: there is a ∼10% increase in PO_2_F_2_^−^ concentration and ∼19% increase in HF concentration with 0.1 mM Mn^2+^ added, and a ∼30% increase in PO_2_F_2_^−^ concentration and ∼59% increase in HF concentration with 1.0 mM Mn^2+^ added, even with potential underestimation of degradation species at 1.0 mM due to signal broadening. The apparent dose–response relationship confirms that at least some of the degradation enhancement is specifically due to the Mn(TFSI)_2_, consistent with the relaxometry results in Fig. 7a.

Previous work on electrolytes heated with 0 *versus* 100 or 300 mM Mn^2+^ has shown a dramatic effect of Mn^2+^ on electrolyte degradation, with paramagnetic solutions turning brown after 8 days at 55 °C.^8,14^ It was proposed that Mn^2+^ coordination to PF_6_^−^ facilitated the formation of reactive PF_5_.^8^ (We note that a brown discolouration may also indicate MnO_2_ formation and possibly oxygen/water contamination.) In comparison, the degradation of samples in our work is relatively minor, particularly considering that samples were heated at 60 °C for ten weeks. No difference in colouration was observed between diamagnetic and paramagnetic samples after heating. The EPR and NMR data (Fig. 2 and 5, respectively) clearly show that Mn^2+^ preferentially interacts with PO_2_F_2_^−^ over PF_6_^−^ in the inner coordination shell, which is also confirmed by DFT calculations (Table S2). At small Mn^2+^ concentrations, such as those used in this work, the probability that PF_6_^−^ enters the Mn^2+^ inner coordination shell once Mn^2+^ is surrounded by PO_2_F_2_^−^ may be reduced significantly, consistent with the ^19^F NMR studies. Thus, the PF_6_^−^ decomposition effect may become self-limiting, especially in cases where LiPO_2_F_2_ is used as an electrolyte additive. At the large Mn^2+^ concentrations used in previous studies, far more PO_2_F_2_^−^ would be required to coordinate all Mn^2+^, and complete coordination to PO_2_F_2_^−^ may not be possible, resulting in “free” Mn^2+^ and far more electrolyte degradation. The dissolved Mn^2+^ concentration in cells is generally small as Mn^2+^ migrates to the negative electrode and deposits there; furthermore, when dissolution occurs *in situ* into an already-degraded electrolyte, PO_2_F_2_^−^ may already exist in solution, which is not the case when Mn^2+^ model compounds are added to pristine electrolyte. Therefore, while the results in Fig. 7 are consistent with previous observations of Mn^2+^-induced electrolyte degradation, it also appears that this effect is unlikely to be a main contributor to electrolyte degradation in cells, with most of the harm of dissolved transition metals occurring once they are deposited at the anode. These results also show that NMR relaxometry may be used in these systems as a probe for electrolyte degradation, as long as the concentration of paramagnetic ions is constant; this method may also be applied in other systems, if a degradation product coordinates more closely to the transition metal ions than to the pristine electrolyte species.

#### ICP-OES experiments of transition metal metathesis

Our studies of transition metal coordination to a wider range of anionic species in solution were complicated by the low solubility of many lithium salts, as well as the finding that the addition of many lithium salts induces precipitation of transition metals. Thus, the effect of a large series of lithium salts—some of which have been shown to be present in the SEI—on transition metal solubility was investigated.

**Fig. 8:**
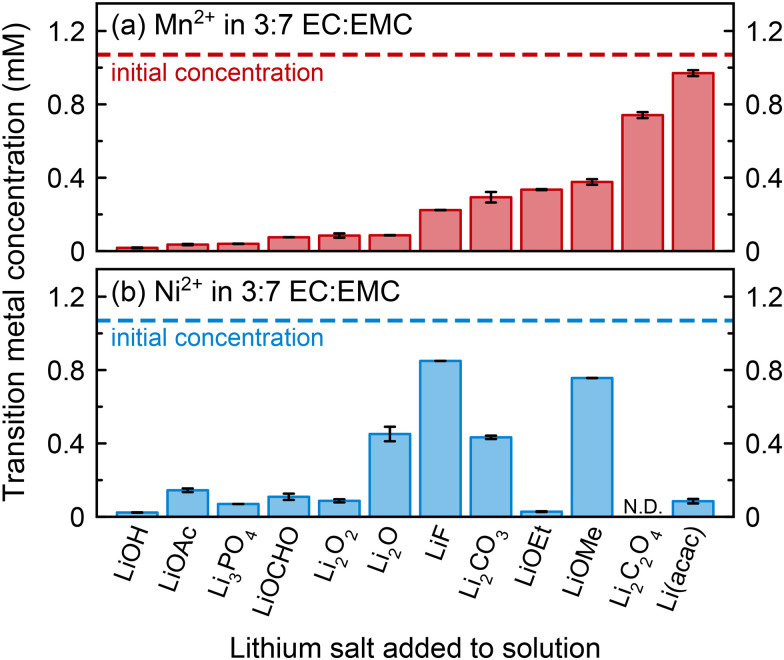
(a) Mn^2+^ or (b) Ni^2+^ concentrations in 3 : 7 EC : EMC (v/v) solutions after lithium salts were added to solutions containing ∼1 mM of Mn(TFSI)_2_ or Ni(TFSI)_2_. The initial concentration is indicated by the dashed line (1.07 mM for both Mn^2+^ and Ni^2+^); bars indicate the new transition metal concentration two days after lithium salt addition. All concentrations were measured by ICP-OES. Error bars show the range obtained for duplicate experiments (*i.e.*, measurements on two different solutions).

Fig. 8 shows ICP-OES results for the concentration of Mn^2+^ or Ni^2+^ in 3 : 7 EC : EMC (v/v) after 1 mM solutions were shaken with various lithium salts and stored for two days. All lithium salts studied induced some amount of transition metal precipitation, including the organic lithium salts, suggesting that metals may be able to precipitate directly in both the outer organic SEI and the inner inorganic SEI. The addition of Li_2_O_2_ to Mn^2+^-containing solution resulted in the solution immediately turning brown in colour, likely due to the oxidation of Mn^2+^ to yield the brown MnO_2_ precipitate. The largest differences between Ni^2+^ and Mn^2+^ precipitation behaviour were observed with Li_2_O, LiF, and LiOMe, where more Mn^2+^ precipitation occurred, and with LiOEt and Li(acac), where more Ni^2+^ precipitation occurred. Generally, this seems to suggest a possible preference for Mn^2+^ precipitation with harder, more inorganic components, and Ni^2+^ precipitation with softer, more organic components, which is consistent with the classification of Mn^2+^ as a hard cation and Ni^2+^ as borderline.^118,119^ This may explain why some studies that have examined metals both in the electrolyte solution and on the anode surface have shown that a greater fraction of dissolved Mn^2+^ is deposited compared to dissolved Ni^2+^ or Co^2+^.^41,120^ Interestingly, with Li(acac), not only did ∼91% of Mn^2+^ remain in solution, but the lithium concentration in solution increased from 0.474 ± 0.004 mM when Li(acac) was stored in a diamagnetic solution to 3.43 ± 0.02 mM when Li(acac) was stored in a Mn^2+^-containing solution; *i.e.*, an additional 2.96 mM Li dissolved, or ∼3 : 1 Li : Mn ratio. This enhanced Li dissolution suggests that Li/Mn exchange occurs, and Mn^2+^ chelation by acac^−^ is favourable, but the product is soluble. This ICP-OES result also supports the interpretation of the relaxometry results in Fig. 6b.

While previous studies have exposed transition metal ions to cycled graphite anodes,^73,75–79^ specific learnings from those experiments are complicated by the fact that the exact SEI composition is unknown, and it is therefore unknown which species the transition metal ions are exchanging with. We also note that the exact nature of an SEI greatly depends on the specific electrolyte composition used and particularly any SEI additives employed. This experiment expands on that previous work, as well as previous work studying *ex situ* exchange with some inorganic lithium salts,^39,74,75^ revealing that transition metals can exchange with many different species, including organic salts.

The ICP-OES results show that various SEI salts can induce transition metal precipitation out of EC : EMC solution, lending credence to metathesis deposition mechanisms. (We note that this is not necessarily mutually exclusive with possible reduction mechanisms; the first step for any reduction that may occur is initial incorporation into the SEI, and one such mechanism occurs *via* ion exchange.) However, when metals are surrounded by suitably coordinating species in a degraded electrolyte solution, such as PO_2_F_2_^−^/HPO_2_F_2_, desolvation and precipitation at the anode surface may become less favourable. More exchange-type experiments that are explicitly conducted in the presence of degradation species such as PO_2_F_2_^−^ may help determine the realistic likelihood of transition metal metathesis. The combined EPR, NMR, and ICP-OES results in this work present a spectrum of coordination behaviour that influences transition metal deposition. With species like PF_6_^−^, Mn^2+^ coordination is not preferred; with species like PO_2_F_2_^−^ or acac^−^, Mn^2+^ coordination is preferred but the complexes are soluble; and with species like PO_4_^3−^ or OH^−^, Mn^2+^ precipitates almost completely. The location where dissolved transition metals ultimately precipitate will likely depend on which species are nearby: if a favourable species encouraging precipitation is present when Mn^2+^ dissolves, it will more likely deposit on the cathode or separator; otherwise, Mn^2+^ may bind to species in solution and either remain in solution or deposit on the anode, depending on the strength and favourability of coordination. A clearer understanding of transition metal complexation behaviours in battery electrolytes may facilitate strategies that prevent transition metal dissolution, encourage precipitation of metal ions at the cathode, and/or enable sequestration of metal ions in solution, ultimately preventing deposition and harmful reactions at the anode.

## Conclusions

Paramagnetic solution NMR and pulsed one and two-dimensional EPR and ENDOR were used to probe the coordination of Mn^2+^ and Ni^2+^ in degraded electrolytes, probing the electron–nuclear interactions from two perspectives. Heat-degraded electrolyte solutions, or solutions containing simulated degradation species, were examined to unravel transition metal coordination to specific solution components of interest. Although in pristine electrolyte solutions the solvation shell is mainly comprised of the solvent molecule EC, the transition metal solvation shell is considerably changed in degraded electrolyte solutions; these results illustrate the power of this joint approach in determining the structures of metal complexes in pristine and degraded electrolytes.

Pulsed EPR experiments of frozen solutions show that in heat- and water-degraded LiPF_6_ electrolytes, Mn^2+^ is coordinated to a combination of anionic and protonated fluorophosphate species. This is consistent with measurements of NMR relaxation rates showing that EC and PF_6_^−^ are increasingly displaced in the Mn^2+^ and Ni^2+^ inner and outer coordination spheres when electrolytes are heated. When water or D_2_O is added to pristine, non-degraded electrolyte solutions containing either LiPF_6_ or LiTFSI salts, EPR experiments show that it enters the Mn^2+^ first coordination sphere and displaces EC to form [Mn(OH_2_)_6_]^2+^/[Mn(OD_2_)_6_]^2+^; however, in LiPF_6_-containing electrolytes, the protonated fluorophosphate species that arise from PF_6_^−^ hydrolysis, specifically HPO_2_F_2_, ultimately displace the water from the first coordination shell. The electron-^1^H hyperfine coupling tensors obtained by simulations of the ENDOR experiments indicate a Mn–H distance of between 3.7 and 4.8 Å, consistent with an HPO_2_F_2_ inner sphere complex. EPR and NMR of solutions containing added LiPO_2_F_2_ complement the results from heated electrolyte solutions, indicating that PO_2_F_2_^−^ is present in both inner sphere and outer sphere Mn^2+^ complexes.

While EPR experiments of frozen solutions allow for a more detailed structural analysis of Mn^2+^ complex formation, the use of NMR relaxometry permits a more rapid screening of the effect of additives and electrolyte degradation on complex formation. For example, NMR experiments showed that the addition of ethylene glycol to the electrolyte causes the displacement of EC in the first solvation shell of Mn^2+^ and Ni^2+^ (as seen for PO_2_F_2_^−^), now also reducing the size of the first solvation shell. Addition of the larger acetylacetonate ligand displaced both EC and PF_6_^−^. Examination of samples heated with and without Mn^2+^ indicated that dissolved transition metals likely do not contribute significantly to decomposition of the bulk electrolyte solution in the period after dissolution and before deposition, as has been previously suggested. This is because transition metals are present in small concentrations and coordination to typical electrolyte degradation species is preferred over coordination to, and decomposition of, pristine electrolyte components. Lastly, ICP-OES experiments targeting the strength and extent of transition metal coordination showed that many lithium-containing salts proposed to be present in the SEI appear to induce some amount of transition metal exchange, typically associated with precipitation of the corresponding transition metal salts.

This work demonstrates the importance of considering degradation species in the transition metal solvation shell, which must be considered when designing representative experimental model systems and when performing computational work. This work also supports the metathesis mechanism of transition metal incorporation into the SEI, while identifying differences in how Mn^2+^ and Ni^2+^ are desolvated, which may affect the likelihood of metal deposition and the activity of deposited metals towards electrolyte decomposition. The phenomenon of transition metal coordination to solution degradation species that is shown in this work may present an additional, largely overlooked mechanism for dissolution behaviour. New insights about transition metal coordination may be leveraged to reduce transition metal dissolution and deposition, preventing battery degradation and extending lifetime.

## Conflicts of interest

There are no conflicts to declare.

## Supplementary Material

EE-018-D5EE01250C-s001

EE-018-D5EE01250C-s002

EE-018-D5EE01250C-s003

## Data Availability

In addition to the NMR, EPR, and DFT figures and tables in the supplementary information (SI), we include ZIP files with further supporting data for EPR and DFT studies of Mn coordination. These include CSV files with hyperfine coupling constants and XYZ files with final structures of all complexes. Supplementary information: Further DFT calculations, experimental and simulated EPR spectra, and NMR relaxometry. See DOI: https://doi.org/10.1039/d5ee01250c.
