# Supplementary material for: Transition metal coordination to degradation products in battery electrolytes revealed by NMR and EPR spectroscopy
Source: Energy Environ Sci. 2025 Oct 29;18(23):10147–63. doi: 10.1039/d5ee01250c (PMC12569987; doi:10.1039/d5ee01250c)
Supplement: EE-018-D5EE01250C-s003 [file EE-018-D5EE01250C-s003.pdf]

Supplementary Information (SI) for Energy & Environmental Science.
This journal is © The Royal Society of Chemistry 2025

Transition metal coordination to degradation products in battery electrolytes revealed by NMR and EPR spectroscopy

Jennifer P. Allen, Conrad Szczuka, Erlendur Jónsson, Rüdiger-A. Eichel, Josef Granwehr, Clare P. Grey

Supplementary Information

EPR

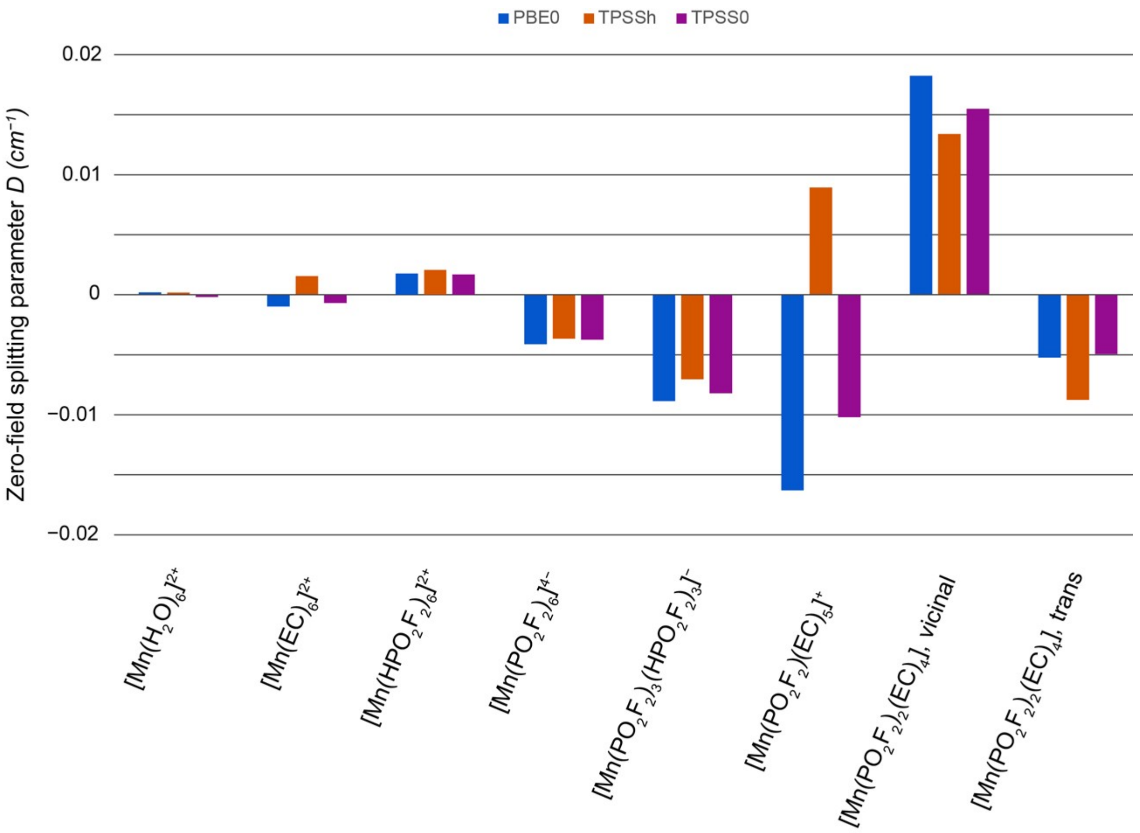

Figure S1. Zero-field splitting parameters for Mn^{2+} complexes calculated from DFT using the functionals PBE0, TPSSh, and TPSS0 which exhibit a Hartree-Fock exchange of 25, 10, and 25 %, respectively. Scalar relativistic corrections (ZORA), extended and partially decontracted basis sets, spin–orbit mean-field approximation (SOMF(1X) command), and quasi-relativistically modified expressions for the property operators (picture-change effects) were applied, as described in the Experimental section of the main text. We note that the calculation of zero-field splitting parameters is still challenging, particularly for d^5 systems where several many-electron integrals need to be accurately solved to adequately address spin–spin and spin–orbit coupling contributions. These two contributions of similar order of magnitudes but opposing signs result in comparably small values for the zero-field splitting. This effect explains the sign differences when using different functionals for the analysed structures. In more detail, the percentage of Hartree-Fock exchange is seemingly affecting D more than the choice of DFT functional. Nonetheless, the overall trend in magnitude gives a useful estimate to correlate with experimental field-swept Hahn-echo EPR spectra.

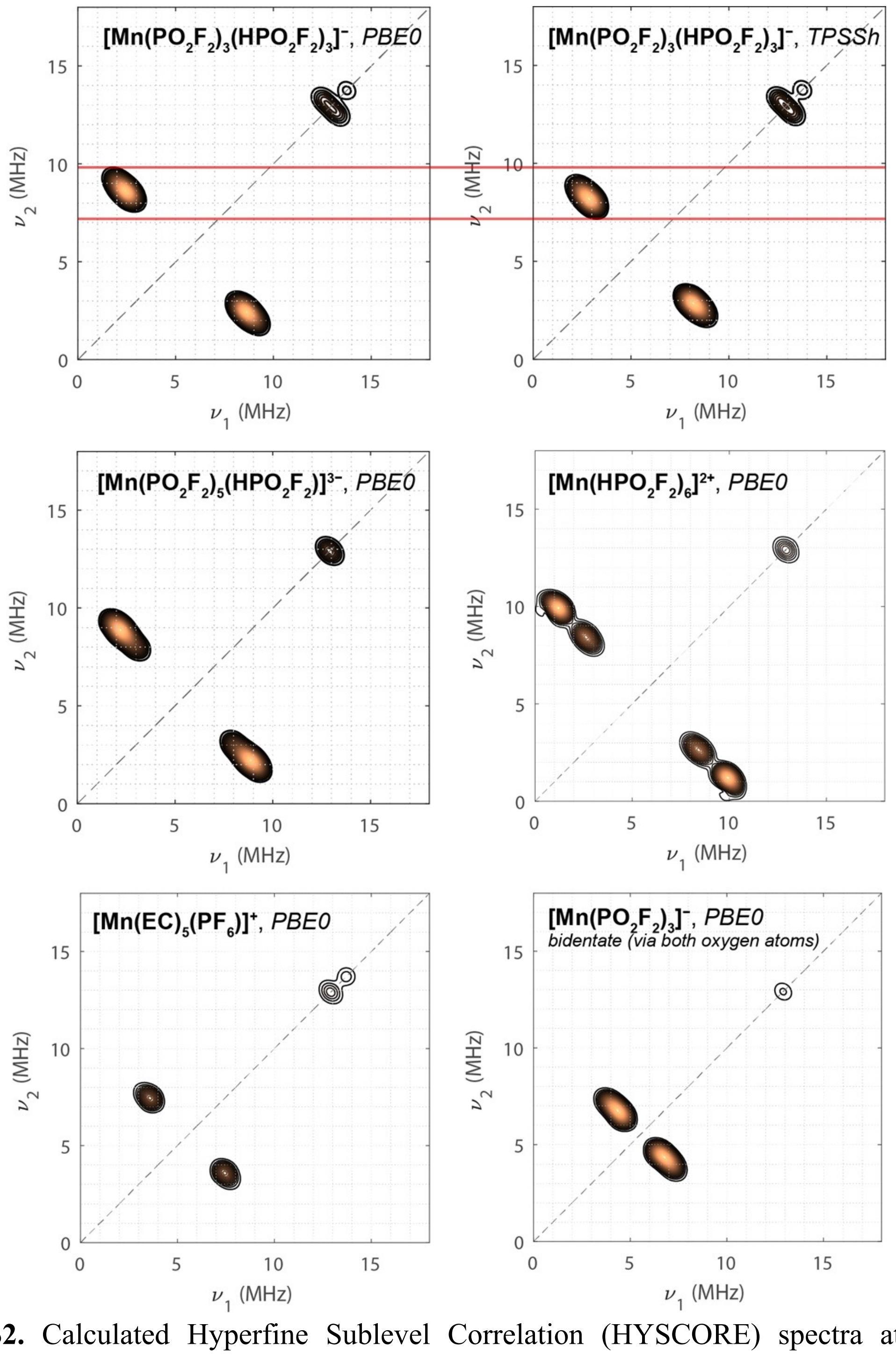

Figure S2. Calculated Hyperfine Sublevel Correlation (HYSCORE) spectra at X-band frequencies. Mn^{2+} complexes, coordination specifics, and the DFT functional are specified. HYSCORE simulation parameters mimic the experimental acquisition parameters as stated in the Experimental section of the main text using a static magnetic field of 325 mT. Red lines are a guide to the eye. In the top row, two DFT functionals are compared. PBE0 exhibiting Hartree-Fock exchange of 25 % leads to a roughly 0.3 MHz larger ^{31}P hyperfine coupling than TPSSh exhibiting 10 % of Hartree-Fock exchange. In the middle row, alternative structure proposition including one or six ^{1}H atoms are shown. In the lower row, HYSCORE spectra of plausible alternatives based on the available chemistry in the experimental samples are plotted. These spectra do not reflect the experimental spectra shown in Figure 2. It is noted that the fictitious ^{19}F that is directly bonded to the Mn would appear in the second quadrant as a signal in the so-called strong coupling regime.

Table S1. Experimental and DFT-calculated isotropic ^{55}Mn hyperfine coupling constants. For DFT calculations, the applied functional is indicated. Using either PBE0 or TPSSh functionals, deviations of $A(^{55}Mn)$ are around 25 MHz.

Complex	Experiment (MHz)	DFT, PBE0 (MHz)	DFT, TPSSh (MHz)
$[Mn(H_2O)_6]^{2+}$, *ref.* [1]	± 252		
Mn^{2+} in $LiPF_6$ electrolyte, *as prepared*	± 273		
Mn^{2+} in $LiPF_6$ electrolyte + 1 vol.% H_2O, *as prepared*	± 273		
Mn^{2+} in $LiPF_6$ electrolyte, *heated at 35 °C for 24 h*	± 273		
$[Mn(H_2O)_6]^{2+}$		−214	−192
$[Mn(EC)_6]^{2+}$		−214	−190
$[Mn(HPO_2F_2)_6]^{2+}$		−217	−190
$[Mn(PO_2F_2)_3(HPO_2F_2)_3]^-$		−213	−190

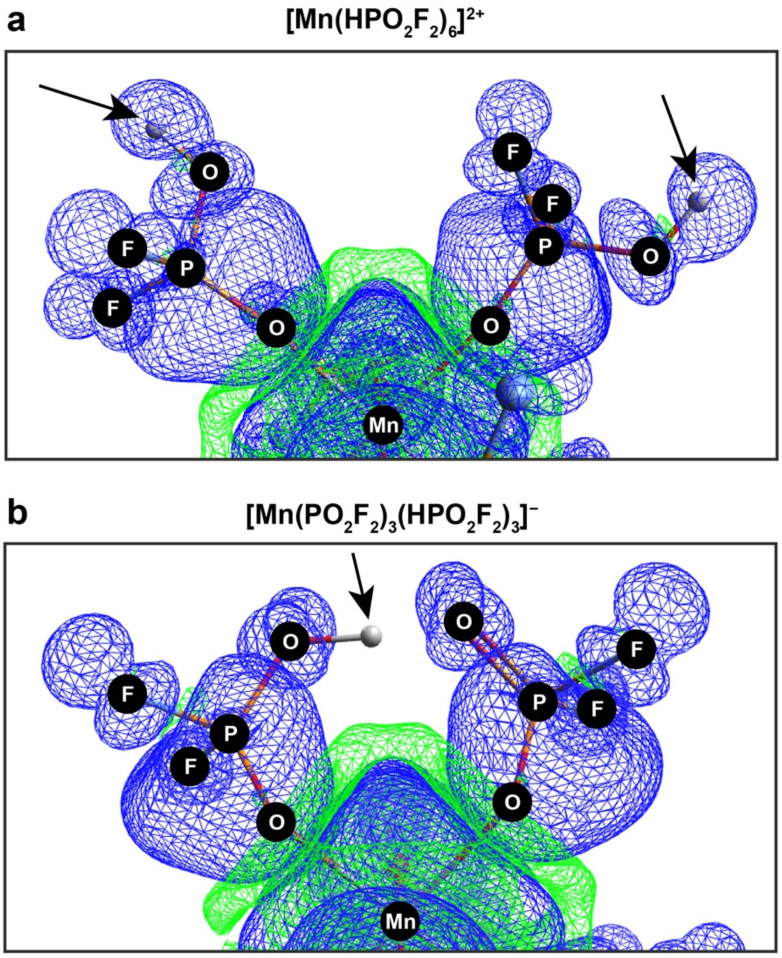

Figure S3. Spin density plot of Mn-fluorophosphate complexes. The structures were geometry optimised on a DFT/TPSSh/def2-TZVP(-f) level of theory. Incorporation of one solvation shell leads to an energy minimum when OH groups face outwards for $[Mn(HPO_2F_2)_6]^{2+}$ (a) and when O-H-O hydrogen bonds are formed for $[Mn(PO_2F_2)_3(HPO_2F_2)_3]^{-}$. The spin density is displayed at an iso-value of 0.00002 with positive values in blue and negative in green. For clarity, relevant nuclear positions were retroactively emphasised and hydrogen nuclei are pointed at by arrows. The distance between the ^{55}Mn and ^{1}H nuclei is 5.0 Å and 5.5 Å (a) and 3.9 Å (b). A large dipolar hyperfine interaction is partially shielded by the negative spin density. This negative spin density contribution is not spherically symmetric, so its contribution depends on the location of the H atom on the coordination shell, which is possibly also affected by molecules in outer solvation spheres that have not been accounted for here.

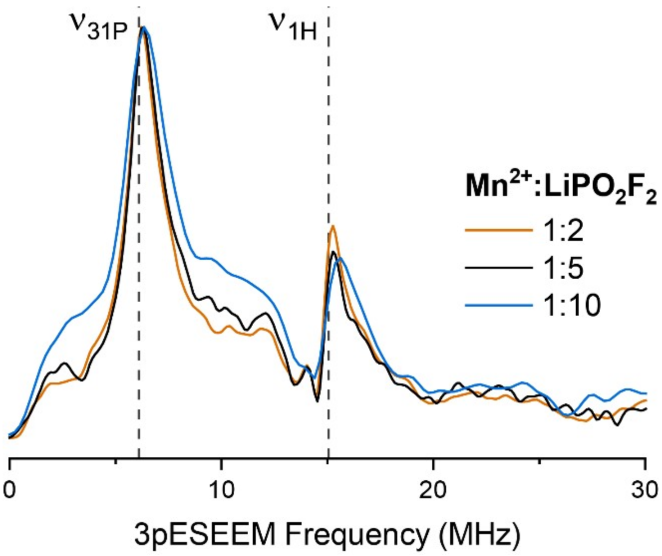

Figure S4. Three-pulse electron spin echo envelope modulation (ESEEM) spectra at X-band frequencies of a solution containing 8 mM Mn^{2+}, 16−80 mM $LiPO_2F_2$, and 1 M $LiPF_6$ in 3:7 EC:EMC. An experimental τ value of 120 ns was used. Spectra are normalised to the maximum intensity. The Mn^{2+}:$LiPO_2F_2$ ratio is given in the legend. Three-pulse ESEEM experiments approximately correspond to HYSCORE spectra projected to one axis, enabling a comparison of the relative contributions from weakly coupled ^{31}P appearing at ν_{31P} and strongly coupled ^{31}P appearing at roughly 1–5 MHz and 9–13 MHz. With increasing concentration of $LiPO_2F_2$, the amount of strongly coupled ^{31}P (tentatively assigned to Mn-O-P fragments) significantly increases compared to more remote ^{31}P contributions. Additionally, the signal at ν_{1H} seems to broaden slightly, which would implicate a stronger hyperfine coupling to ^{1}H, but the effect is too small to draw firm conclusions.

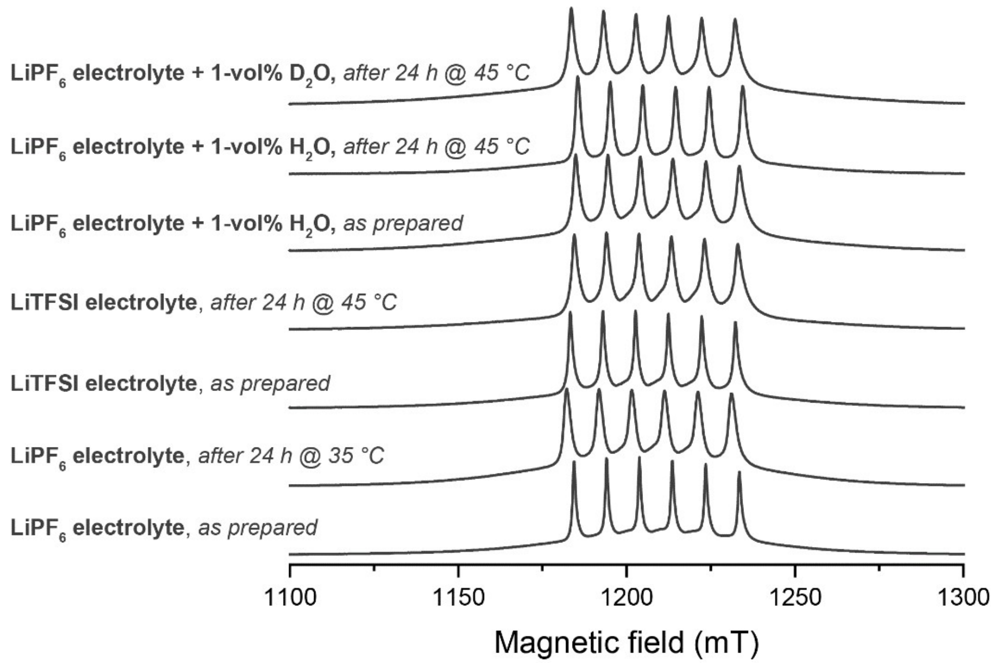

Figure S5. Field-swept echo-detected pulsed EPR spectra at Q-band microwave frequencies. ENDOR measurements in Figure 3 were performed at a magnetic field position corresponding to the low-field maximum. Samples of 8 mM $Mn(TFSI)_2$ dissolved in 3:7 EC:EMC with 1 M salt as indicated and optional heat-treatment or water addition are investigated. Peak position variability results largely from slightly different applied microwave frequencies.

Deuterated solvents

Our previous studies of solution NMR peak broadening showed that ^{19}F peak widths of paramagnetic solutions are far more broad when using CD_3CN than when using d_6-DMSO as a solvent (with CD_3OD in the middle), which we suggested was due to a difference in their Mn^{2+}-solvating abilities.[2] Using an excess of different deuterated solvents, the removal of ^{19}F and ^1H resonances in the ENDOR spectra serves as a gauge of how complete the ligand exchange is. For the tested solvents, carbonate replacement efficiency follows the series $CD_3OD \approx d_6$-DMSO $\gg CD_3CN$, fluorophosphate replacement efficiency follows $CD_3OD > d_6$-DMSO $\gg CD_3CN$ (Figure S6), similar to thermodynamic data of the associated Mn(II) complexes.[3] The large difference between the replacement efficiency with d_6-DMSO and CD_3CN suggests that the previous favourable NMR results with d_6-DMSO were indeed due to displacement of bound NMR species, resulting in narrower peaks.

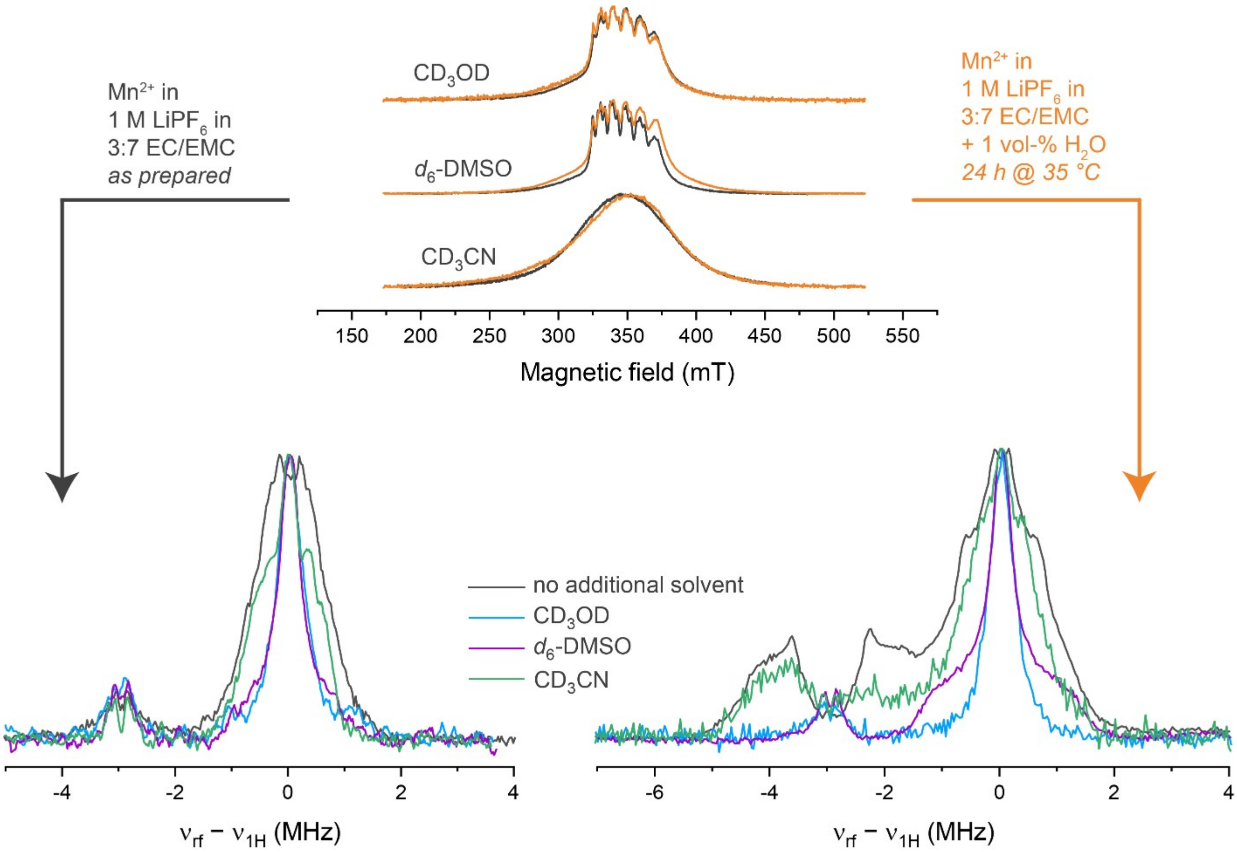

Figure S6. X-band field-swept echo-detected EPR spectra and Q-band ENDOR spectra of 10 vol.% of the indicated samples (and as described in the main text) in deuterated solvents CD_3OD, d_6-DMSO, and CD_3CN. Broadening of EPR spectra likely results from an increase in zero-field splitting parameters and/or in distribution thereof (Figure S1). ENDOR spectra exhibit partial or complete removal of carbonate or fluorophosphate ligands from the first coordination sphere. Residual signal at ν_{1H} corresponds to outer solvation shells and signal centered at ν_{19F} likely to a fluorine-containing anion forming a solvent-separated ion pair. A partial ligand exchange in CD_3CN solutions might explain the large width of the X-band EPR spectrum because the variety of ligands results in the largest deviation from octahedral symmetry.

NMR

Paramagnetic relaxation theory

Solomon-Bloembergen-Morgan theory[4–8] provides a framework to understand the nuclear relaxation behaviour driven by paramagnetic species. Equations 2 and 3 describe the longitudinal and transverse relaxation rates, or R_{1M} and R_{2M}, respectively, of nuclei that are bound to a paramagnetic metal ion. The relaxation rate *R* is simply the inverse of the measured relaxation time *T*. These equations comprise two terms, a dipolar term and a contact term. The dipolar term arises from the Solomon equations[6] and describes through-space coupling of the nuclear and electron spin; the contact term arises from the Bloembergen equations[7] and describes the electron density at the nucleus.[4,5] The other terms in Equations 2 and 3 are as follows: permeability of a vacuum, μ_0; nuclear gyromagnetic ratio, γ_I; electron spin g-factor, g_e; Bohr magneton, μ_B; electron spin, *S*; distance between the nucleus and paramagnetic ion, *r*; correlation time for the dipolar term, τ_c^{dip}; Larmor frequencies for the nuclear spin, ω_I, and for the electron spin, ω_S; hyperfine interaction constant, $A/\hbar$ in rad·s^{-1}, or A/h in MHz; and correlation time for the contact term, τ_c^{con}.

$$R_{1M} = \frac{2}{15}\left(\frac{\mu_0}{4\pi}\right)^2\frac{\gamma_I^2 g_e^2 \mu_B^2 S(S+1)}{r^6}\left(\frac{3\tau_c^{dip}}{1+\omega_I^2(\tau_c^{dip})^2}+\frac{7\tau_c^{dip}}{1+\omega_S^2(\tau_c^{dip})^2}\right)+\frac{2S(S+1)}{3}\frac{A^2}{\hbar^2}\left(\frac{\tau_c^{con}}{1+\omega_S^2(\tau_c^{con})^2}\right)$$

Equation 2

$$R_{2M} = \frac{1}{15}\left(\frac{\mu_0}{4\pi}\right)^2\frac{\gamma_I^2 g_e^2 \mu_B^2 S(S+1)}{r^6}\left(4\tau_c^{dip}+\frac{3\tau_c^{dip}}{1+\omega_I^2(\tau_c^{dip})^2}+\frac{13\tau_c^{dip}}{1+\omega_S^2(\tau_c^{dip})^2}\right)+\frac{1S(S+1)}{3}\frac{A^2}{\hbar^2}\left(\text{[illegible]} \right.$$

Equation 3

For Mn^{2+}, because it has a relatively slow electronic relaxation time,[4] and because ω_S is very large,[7,9,10] the $1/\omega_S^2\tau_c^2$ terms in Equations 2 and 3 are small.[7,9,11] We can therefore approximate R_{1M} as purely dipolar, while R_{2M} contains both dipolar and contact terms; thus, if the R_{2M} contact term is very large, then the transverse relaxation rate may become much larger than the longitudinal relaxation rate, $R_{2M} >> R_{1M}$.

The correlation time τ_c^{dip} is determined by whichever is fastest among the molecular rotation correlation time τ_r, the electronic relaxation time τ_e, and the chemical exchange time τ_M. The

correlation time τ_c^{con} arises from the isotropic Fermi contact interaction; hence, it is determined by only τ_e and τ_M.

$$(\tau_c^{dip})^{-1} = \tau_r^{-1} + \tau_e^{-1} + \tau_M^{-1} \qquad \text{Equation 4}$$

$$(\tau_c^{con})^{-1} = \tau_e^{-1} + \tau_M^{-1} \qquad \text{Equation 5}$$

It is therefore possible to have a short τ_r—as is the case for low-viscosity electrolytes comprised of small molecules—and a significantly longer τ_e and τ_M, resulting in $\tau_c^{con} >> \tau_c^{dip}$. If τ_e and τ_M are slow, then the contact term may become large and $R_{2M} >> R_{1M}$.[7,12] A large hyperfine interaction may also result in a large contact term and $R_{2M} >> R_{1M}$, which may occur when the paramagnetic ion coordinates in an environment closer to the nucleus of interest.[13] Comparison of the transverse and longitudinal relaxation rates may therefore provide insight into the structure and dynamics of a paramagnetic ion's solvation shell.

The total measured nuclear relaxation rates comprise a bulk diamagnetic component and an inner sphere paramagnetic component. The longitudinal paramagnetic relaxation enhancement, or R_{1p}, is described in Equation 6, where R_1 is the measured relaxation rate, R_{1d} is the diamagnetic relaxation rate, and R_{1M} is the relaxation rate of nuclei bound to paramagnetic centres, described in Equation 2.[4,13] The paramagnetic relaxation enhancement is proportional to f_M, which represents the fraction of nuclei coordinating to the paramagnetic metal ion. If the metal concentration or solvation number increases, f_M will increase, as more nuclei will be coordinated to metal ions; if the concentration of the nucleus being probed increases, f_M will decrease, as more nuclei are free and the bound fraction is lower. In the samples explored here, most variables are kept constant, so that we may interpret whether the bound fraction of a solution species is changing, *e.g.*, if less EC is bound to Mn^{2+} when $PO_2F_2^-$ is added.

$$R_{1p} = R_1 - R_{1d} = f_M \frac{1}{(1/R_{1M}) + \tau_M} \qquad \text{Equation 6}$$

Equation 6 also contains a τ_M term, the chemical exchange time, which indicates the lifetime of the bound complex. This model presents relaxation as arising from a bulk diamagnetic component and an inner sphere paramagnetic component; the chemical exchange of species

within the metal coordination sphere controls the size of the inner sphere paramagnetic component. Relaxation in the second, outer sphere is a lesser component[5] and is generally neglected here: as second sphere species are not in contact with the paramagnetic metal ion, second sphere relaxation is purely dipolar. While it is difficult to know its size without targeted experiments, we estimate ~10% of the overall relaxation[4] is a plausible second sphere component. The role of the second coordination sphere is discussed below in the context of Figure 6a.

Deuterated solvents

We have previously shown that d_6-DMSO is an effective agent to reduce paramagnetic peak broadening,[2] presumably by coordinating to the paramagnetic ions. To explore this phenomenon further, the ^{1}H EC and ^{19}F PF_6^- relaxation rates of Mn^{2+}-containing electrolyte solutions were measured when either CD_3CN, MeOD, or d_6-DMSO was added (Figure S7). With CD_3CN and MeOD addition, relaxation rates do not change significantly. With d_6-DMSO addition, the ^{1}H EC R_{1p} values decrease and the ^{19}F PF_6^- R_{1p} values remain constant; by contrast, the ^{1}H EC R_2/R_1 ratio remains constant and the ^{19}F PF_6^- R_2/R_1 ratio decreases.

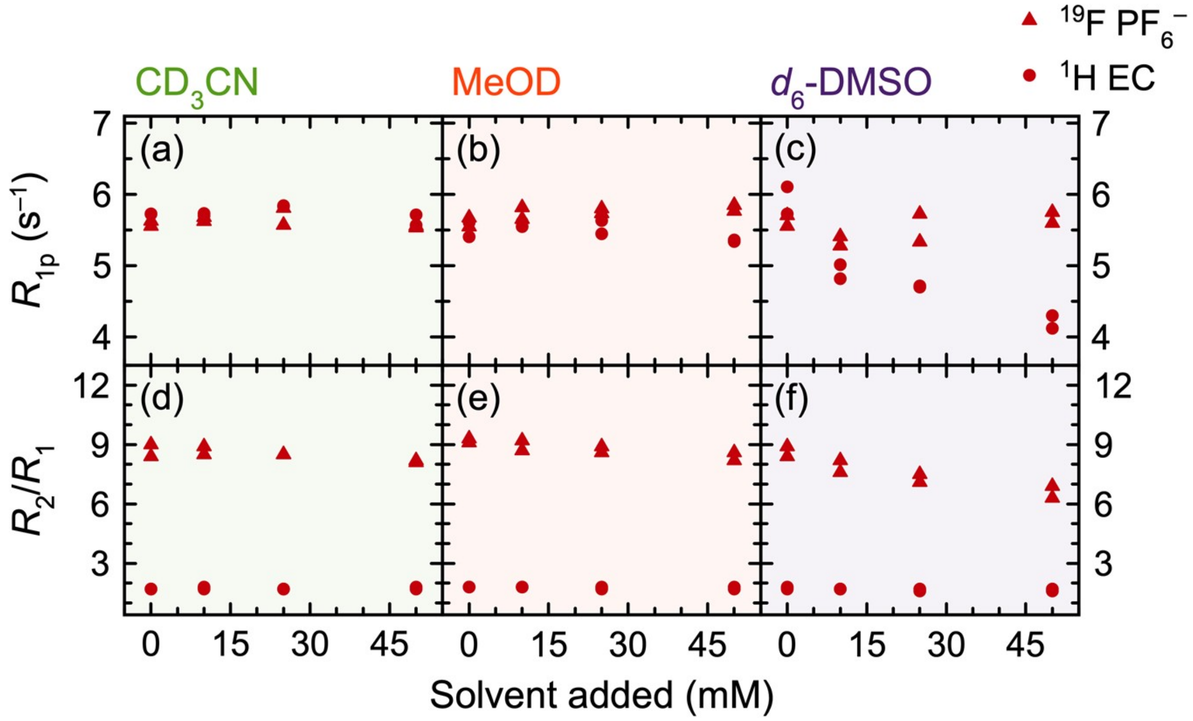

Figure S7. (a–c) R_{1p} values and (d–f) R_2/R_1 ratios for ^{19}F PF_6^- resonances (triangles) and ^{1}H EC resonances (circles) in electrolyte solutions containing 1 mM $Mn(TFSI)_2$ with 0–50 mM CD_3CN, MeOD, or d_6-DMSO added. Electrolyte solution comprises 1 M $LiPF_6$ in 3:7 EC:EMC (v/v).

Unlike addition of CD_3CN or MeOD, addition of d_6-DMSO to Mn^{2+}-containing electrolyte solution significantly decreases the ^{1}H EC R_{1p} values. This suggests that EC is replaced in the Mn^{2+} solvation shell by d_6-DMSO, resulting in a smaller fraction of EC coordinated to Mn^{2+}. Interestingly, the ^{19}F PF_6^- R_{1p} values remain constant, while the ^{19}F PF_6^- R_2/R_1 ratio decreases from ~9 to ~7 upon d_6-DMSO addition (Figure S7f). This convergence of R_2 and R_1 may indicate a change in the fraction of inner sphere versus outer sphere PF_6^- and a change in the correlation times governing relaxation. For example, if the R_2 relaxation is dominated by an exchange effect, this could potentially indicate an increase in the rate of PF_6^- exchange. The overall effect of d_6-DMSO dilution of NMR samples may differ for neutral molecules and anionic species. The beneficial effect of d_6-DMSO on the linewidths of neutral species would likely fade if the solvent (or degradation species) is a better donor than d_6-DMSO, and therefore resists displacement by d_6-DMSO. The beneficial effect on linewidths of anionic species would likely fade if the anion is a better donor than d_6-DMSO, or if the R_2 value is small. The more severe spectral broadening observed with CD_3CN over MeOD, despite both having little or no effect on Mn^{2+} coordination, suggests that it may be a different effect moderating the broadening severity, such as a τ_r effect where the greater viscosity of MeOD versus CD_3CN permits faster relaxation and narrower peaks. The beneficial effect of d_6-DMSO towards regaining spectral resolution in Mn^{2+}-containing samples is complex and determined not only by the fraction of the species in the solvation shell but also by the relevant correlation times; how a given degradation species is affected by d_6-DMSO will vary based on its role in the Mn^{2+} solvation shell.

DFT

Ion-pairing

The ion-pairing of Mn^{2+} with the three key anions tested here, PF_6^-, $PO_2F_2^-$, and $TFSI^-$, was studied with DFT. Ion-pairs were generated programmatically to explore the configurational space of the complexes. For Mn^{2+}, one or two anions were used, so that the complexes explored were: $Mn(PF_6)^+$, $Mn(PF_6)_2$, $Mn(TFSI)^+$, $Mn(TFSI)_2$, $Mn(PO_2F_2)^+$ and $Mn(PO_2F_2)_2$. Both c1 and c2 conformers of $TFSI^-$ were used.

In each case, the structures had their geometry optimised with the B3LYP functional (D3BJ dispersion correction) with the def2-TZVPP basis set. Orca version 5.0.3 was used throughout this section along with the default RIJCOSX settings. Furthermore, the tight SCF and geometry optimisation convergence criteria was used. After optimisation, frequency calculations were performed to confirm convergence and get thermochemistry results. The resulting ion-pair dissociation energies are shown in Table S2.

Table S2. The ion-pair dissociation energies, ΔE_d and ΔG_d, calculated for the Mn-complexes. The energies were calculated with: $\Delta E_d = E_{Mn} + n{*}E_{anion} - E_{ion\text{-}pair}$.

Complex	ΔE_d [kJ/mol]	ΔG_d [kJ/mol]
$Mn(PF_6)^+$	1322.2	1285.8
$Mn(PF_6)_2$	2028.1	1933.6
c1-$Mn(TFSI)^+$	1419.7	1378.5
c2-$Mn(TFSI)^+$	1417.2	1376.5
c1c1-$Mn(TFSI)_2$	2108.6	2003.1
c1c2-$Mn(TFSI)_2$	2109.7	2005.5
c2c2-$Mn(TFSI)_2$	2111.3	2001.5
$Mn(PO_2F_2)^+$	1478.5	1438.3
$Mn(PO_2F_2)_2$	2250.3	2152.9

Examining the ion-pair dissociation energies, both ΔE_d and ΔG_d, (higher number means stronger binding between anion and cation) shows that the $PO_2F_2^-$ binds stronger to the Mn^{2+} than both PF_6^- and $TFSI^-$. This is further confirmation of Figure 5, where small amounts of $PO_2F_2^-$ were able to decrease the paramagnetic effect that Mn^{2+} has on the other anions by binding strongly to it. Comparing the results of the $TFSI^-$ conformers show very little effects on the conformational equilibrium, unlike for the LiTFSI case. For the neutral case, the overall trend still holds with $Mn(PO_2F_2)_2 > Mn(TFSI)_2 > Mn(PF_6)_2$.

References

1. A. R. Coffino and J. Peisach, *J. Magn. Reson., Ser. B*, 1996, **111**, 127–134.
2. J. P. Allen and C. P. Grey, *J. Phys. Chem. C*, 2023, **127**, 4425–4438.
3. H. Konieczna, D. Lundberg and I. Persson, *Polyhedron*, 2021, **195**, 114961.
4. I. Bertini, C. Luchinat, G. Parigi and E. Ravera, *NMR of Paramagnetic Molecules: Applications to Metallobiomolecules and Models*, Elsevier, 2016.
5. A. J. Pell, G. Pintacuda and C. P. Grey, *Prog. Nucl. Magn. Reson. Spectrosc.*, 2019, **111**, 1–271.
6. I. Solomon, *Phys. Rev.*, 1955, **99**, 559–565.
7. N. Bloembergen, *J. Chem. Phys.*, 1957, **27**, 572–573.
8. N. Bloembergen and L. O. Morgan, *J. Chem. Phys.*, 1961, **34**, 842–850.
9. M. R. Jensen and J. J. Led, *J. Magn. Reson.*, 2004, **167**, 169–177.
10. D. Goldfarb and S. Stoll, Eds., *EPR Spectroscopy: Fundamentals and Methods*, John Wiley & Sons, Chichester, UK, 2018.
11. P. Caravan, C. T. Farrar, L. Frullano and R. Uppal, *Contrast Media Mol. Imaging*, 2009, **4**, 89–100.
12. Z. Luz, *Isr. J. Chem.*, 1971, **9**, 293–300.
13. T. J. Swift and R. E. Connick, *J. Chem. Phys.*, 1962, **37**, 307–320.
